# Predicting early extrahepatic recurrence after local treatment of colorectal liver metastases

**DOI:** 10.1093/bjs/znac461

**Published:** 2023-01-19

**Authors:** G E Wensink, Karen Bolhuis, Marloes A G Elferink, Remond J A Fijneman, Onno Kranenburg, Inne H M Borel Rinkes, Miriam Koopman, Rutger-Jan Swijnenburg, Geraldine R Vink, Jeroen Hagendoorn, Cornelis J A Punt, Jeanine M L Roodhart, Sjoerd G Elias

**Affiliations:** Department of Medical Oncology, University Medical Centre Utrecht, Utrecht University, Utrecht, the Netherlands; Department of Medical Oncology, Amsterdam UMC location University of Amsterdam, Amsterdam, the Netherlands; Department of Gastrointestinal Oncology, Netherlands Cancer Institute, Amsterdam, the Netherlands; Department of Research and Development, Netherlands Comprehensive Cancer Organisation (IKNL), Utrecht, the Netherlands; Department of Pathology, Netherlands Cancer Institute, Amsterdam, the Netherlands; Department of Surgery, University Medical Centre Utrecht, Utrecht University, Utrecht, the Netherlands; Utrecht Platform for Organoid Technology, University Medical Centre Utrecht, Utrecht University, Utrecht, the Netherlands; Department of Surgery, University Medical Centre Utrecht, Utrecht University, Utrecht, the Netherlands; Department of Medical Oncology, University Medical Centre Utrecht, Utrecht University, Utrecht, the Netherlands; Department of Surgery, Amsterdam UMC location University of Amsterdam, Amsterdam, the Netherlands; Department of Medical Oncology, University Medical Centre Utrecht, Utrecht University, Utrecht, the Netherlands; Department of Research and Development, Netherlands Comprehensive Cancer Organisation (IKNL), Utrecht, the Netherlands; Department of Surgery, University Medical Centre Utrecht, Utrecht University, Utrecht, the Netherlands; Department of Epidemiology, Julius Centre for Health Sciences and Primary Care, University Medical Centre Utrecht, Utrecht University, Utrecht, the Netherlands; Department of Medical Oncology, University Medical Centre Utrecht, Utrecht University, Utrecht, the Netherlands; Department of Epidemiology, Julius Centre for Health Sciences and Primary Care, University Medical Centre Utrecht, Utrecht University, Utrecht, the Netherlands

## Abstract

**Background:**

Patients who develop early extrahepatic recurrence (EHR) may not benefit from local treatment of colorectal liver metastases (CRLMs). This study aimed to develop a prediction model for early EHR after local treatment of CRLMs using a national data set.

**Methods:**

A Cox regression prediction model for EHR was developed and validated internally using data on patients who had local treatment for CRLMs with curative intent. Performance assessment included calibration, discrimination, net benefit, and generalizability by internal–external cross-validation. The prognostic relevance of early EHR (within 6 months) was evaluated by landmark analysis.

**Results:**

During a median follow-up of 35 months, 557 of the 1077 patients had EHR and 249 died. Median overall survival was 19.5 (95 per cent c.i. 15.6 to 23.0) months in patients with early EHR after CRLM treatment, compared with not reached (45.3 months to not reached) in patients without an early EHR. The EHR prediction model included side and stage of the primary tumour, *RAS/BRAF^V600E^* mutational status, and number and size of CRLMs. The range of 6-month EHR predictions was 5.9–56.0 (i.q.r. 12.9–22.0) per cent. The model demonstrated good calibration and discrimination. The C-index through 6 and 12 months was 0.663 (95 per cent c.i. 0.624 to 0.702) and 0.661 (0.632 to 0.689) respectively. The observed 6-month EHR risk was 6.5 per cent for patients in the lowest quartile of predicted risk compared with 32.0 per cent in the highest quartile.

**Conclusion:**

Early EHR after local treatment of CRLMs can be predicted.

## Teaser

Early extrahepatic recurrence (EHR) after local treatment of colorectal liver metastases has a major impact on prognosis. A prediction model for 6-month EHR was developed and validated internally in a population-based cohort of 1077 patients. Six-month EHR can be predicted with routine clinical information and may help guide clinical decision-making.

## Introduction

Colorectal cancer liver metastases (CRLMs) are the major cause of colorectal cancer-related death^1^. Local treatment of CRLMs without extrahepatic metastatic involvement, such as liver resection, offers the only chance of cure or long-term survival^2–5^. Improved surgical and ablative techniques, optimization of systemic induction treatment, and more lenient eligibility criteria have increased the number of patients undergoing CRLM resection^4,6^. Relapse after local CRLM treatment occurs in up to 75 per cent of patients, often with unresectable recurrences and decreased survival^5,7,8^. Numerous prediction models for (recurrence-free) survival after local treatment of CRLMs exist^9–15^, but these are not widely used to guide decision-making owing to their inability to identify patients with a sufficiently short survival to render local treatment unjustified. Aspects that might contribute to this include suboptimal incorporation of prognostic factors and the use of (recurrence-free) survival as an endpoint.

Recurrence-free survival (RFS) does not discriminate between intrahepatic and extrahepatic recurrences. Patients with liver-limited recurrences may be eligible for repeat local treatment, resulting in long-term survival^8,16–18^. In contrast, a minority of patients with extrahepatic recurrence (EHR) undergo repeated local treatment^18–20^. An early recurrence, usually defined as recurrence developing within 6 months^21,22^, and EHR are independently associated with poor overall survival (OS) in patients receiving local treatment for CRLMs^21–23^. Therefore, local treatment of CRLMs may not be justified in patients who develop early EHR. Being able to predict early EHR may spare patients invasive treatment, and avoid delay in starting systemic treatment that may effectively treat the systemic disease present. In randomized trials^24,25^, in patients receiving local CRLM treatment, no median OS benefit was seen with perioperative systemic therapy. Early EHR estimates potentially could stratify patients who may and who may not benefit from additional perioperative systemic therapy.

Although early EHR after local treatment of CRLMs is of major clinical importance, no prediction models for early EHR exist. Furthermore, novel prognostic factors, such as primary tumour location and *RAS/BRAF^V600E^* tumour mutational status, may aid in better identifying patients at high risk of early EHR. Patients with right-sided primary tumours have a worse prognosis after local treatment of CRLMs, more recurrences at multiple sites, and less repeated local treatment than patients with left-sided primary lesions^26,27^. The presence of *RAS* and *BRAF^V600E^* mutations is associated with a higher recurrence rate of up to 94 per cent, with EHR not amenable to local therapy, and shorter EHR-free survival (EHRFS)^8,28–30^.

This study aimed to develop and internally validate a prediction model that incorporates primary tumour location and *RAS/BRAF^V600E^* mutational status for early EHR following local treatment of CRLMs using a population-based cohort.

## Methods

### Patient cohort

All patients diagnosed with colorectal cancer between 1 May 2015 and 31 December 2016, who underwent local treatment (resection and/or ablation) with curative intent for CRLMs, were identified in the Netherlands Cancer Registry (NCR)^31^. Patients with extrahepatic metastases before resection, R2 liver resections, appendiceal carcinoma, concomitant local liver treatment other than resection or ablation, and without any follow-up information were excluded. The scientific committee of the Netherlands Comprehensive Cancer Organisation (IKNL) approved the research protocol, and the requirement for written informed consent was waived for this study. The study was performed in accordance with the Declaration of Helsinki and reported according to the TRIPOD guidelines^32^.

### Candidate predictor variables

Data were extracted from the NCR including: age, sex, AJCC tumour (T) category, node (N) category of the primary tumour, location of the primary tumour (right or left colon, or rectum), disease-free interval (DFI) between detection of the primary tumour and metastases, size and number of CRLMs, carcinoembryonic antigen (CEA) level before local treatment of CRLMs, type of local treatment, resection margin status (R0 *versus* R1), and perioperative systemic treatment administered. Major resection was defined as resection of at least four liver segments^33^, synchronous disease as DFI of 6 months or less^34^, and perioperative systemic therapy as treatment administered 100 days or less before and/or after local CRLM treatment and initiated before progression of disease. The intent (neoadjuvant or induction) of systemic treatment was not registered. However, as Dutch colorectal cancer guidelines^35^ recommend not administering perioperative systemic therapy to patients with resectable CRLMs, it was assumed that preoperative systemic treatment was given as induction treatment to achieve CRLM resectability. Further assumptions regarding systemic treatment are described in *Table S1*.

Information on *RAS/BRAF^V600E^* mutational status was retrieved from the NCR and the national automated pathological archive (PALGA^36^), determined in daily practice for the primary tumour or metastases at any time during the disease course. Missing *KRAS*, *NRAS*, and *BRAF^V600E^* mutation status was complemented by an additional Sequenom Massarray® (San Diego, CA, USA) mutation analysis of tumour tissue from 250 patients. These 250 additional samples were selected in such a way as to maximize mutation status information for patient subgroups otherwise under-represented, increasing the likelihood of successful multiple imputation^37^.

### Patient outcomes

Follow-up data for recurrences were collected from medical records until 1 May 2020 and survival was obtained by linkage with the municipal population registry on 31 January 2021. OS was defined as the interval between the date of first local treatment for CRLMs until date of death or last follow-up. RFS and EHRFS were calculated as the interval between the date of first local treatment of CRLMs until the date of a RFS or EHRFS event, which was defined as first recurrence of disease or first EHR or death, whichever occurred first, or censored on last date of RFS or EHRFS without an event respectively. If follow-up for recurrences was shorter than follow-up for survival, all survival follow-up beyond the last follow-up for recurrences was discarded for assessment of RFS, EHRFS or OS. In all patients, a minimum of 1-year RFS and 2-year OS follow-up was ensured. All assumptions regarding OS, RFS, and EHRFS are recorded in *Table S1*.

### Statistical analysis

Standard descriptive statistics were used to describe the study population, including median (i.q.r.) for continuous data, and frequency and percentages for categorical variables. Follow-up and patient outcomes were described using (reverse) Kaplan–Meier approaches.

### Prediction model development and performance assessment

Early EHR (within 6 months^21,22^) was defined as the clinically relevant primary endpoint, owing to the poor prognosis in patients with early EHR and lower chance of repeat local treatment, in contrast to patients with liver-only recurrences. The prognostic impact of this primary endpoint was assessed using landmark analysis at 6 months after CRLM treatment.

Based on published recommendations^38^, there were sufficient data to model 17 coefficients. Nine candidate predictors were selected for model development by assessment of a multidisciplinary team based on literature describing previous prediction models and novel prognostic factors^9–12,26,39^. The predictors, including four continuous variables that were modelled non-linearly, were: neoadjuvant systemic treatment, primary tumour location, T category, N category, *RAS/BRAF^V600E^* mutational status, number of liver metastases, size of largest liver metastasis, preoperative CEA level, and DFI. Multiple imputation with multivariate imputation by chained equations^40^ was used to account for missing data.

A prediction model for EHRFS after local treatment of CRLMs was developed using Cox regression, with a time horizon of 12 months to improve the effective sample size, but with a primary evaluation of the model’s performance for EHR within 6 months. The prediction model was developed in the whole cohort, using Akaike information criterion (AIC)-based backward selection in each imputed data set, leading to a primary model including only predictors selected in at least 50 per cent of imputed data sets, which was then refitted in each imputed data set to obtain a pooled model using Rubin’s rules (EHR model). Adjuvant systemic therapy was included in all models using an offset for expected therapeutic efficacy based on the pooled adjuvant systemic treatment effect from published RCTs^24,25^.

Model performance at 6 and 12 months was assessed using calibration plots, discrimination (C-index), time-dependent receiver operator characteristic (ROC) curves, decision curve analysis, and Nagelkerke’s R^2^. Each measure was determined for each imputed data set separately and pooled using Rubin’s rules, incorporating appropriate data-transformation steps. Decision curve analysis was used to assess the net benefit associated with CRLM treatment decisions based on a given threshold value for 6- or 12-month EHRFS probability^41^. To visualize the model’s potential relevance, Kaplan–Meier curves were plotted for EHRFS, RFS and OS, with patients categorized based on quartiles of predicted EHR risk.

Internal validation by 500-fold bootstrap resampling was used, repeating all model-development steps, in each bootstrap sample, to obtain an overoptimism-corrected model (using uniform shrinkage) and C-index. Internal–external cross-validation was applied, including all modelling steps, to evaluate the generalizability of the model based on three geographical regions.

An exploratory analysis was conducted to test whether the prognostic value of *RAS* mutation for EHRFS depended on the administration of preoperative systemic treatment, as reported by others^28,29^, using a multivariable model with a *RAS* × preoperative systemic treatment interaction term.

A more detailed description of the methods is described in the *supplementary material*. Analyses were performed using SPSS^®^ version 25 (IBM, Armonk, NY, USA) and R version 4.0.3 (R Foundation for Statistical Computing, Vienna, Austria) with the following libraries: rms (V6.2-0), pec (V2022.03.0), survival (V3.3-1), mice (V3.14.0), survival ROC (V1.0.3).

## Results

### Patient cohort

All 1105 patients who underwent local treatment (resection and/or ablation) for CRLMs were selected from the NCR for analysis. No follow-up data were available for 11 of the 1105 patients (less than 1.0 per cent). The primary endpoint (early EHR) was available for 1077 patients.

Patient characteristics are summarized in *Table 1*. Overall median age was 66 years, 403 patients (37.4 per cent) were women, 797 (74.0 per cent) presented with synchronous disease, and 256 (23.8 per cent) with a right-sided primary tumour. A total of 427 patients (39.6 per cent) received systemic treatment, and a major liver resection was performed in 198 (18.4 per cent). The *RAS/BRAF* mutation status was available for 701 patients (65.1 per cent), of whom 352 (50.2 per cent) harboured a *RAS* mutation and 19 (2.7 per cent) a *BRAF^V600E^* mutation.

**Table 1:** Characteristics of 1077 Dutch patients with colorectal cancer diagnosed in 2015–2016 who received local treatment for colorectal liver metastases

	No. of patients* (*n* = 1077)
Age (years), median (i.q.r.)	66 (59–72)
Sex ratio (F : M)	403 : 674
**Site of primary tumour**	
ȃRight colon	256 (23.8)
ȃLeft colon	460 (42.7)
ȃRectum	361 (33.5)
**Chemoradiotherapy to primary tumour**	127 (11.8)
**Tumour category†**	
ȃT1	27 (2.5)
ȃT2	126 (11.8)
ȃT3	740 (69.1)
ȃT4	178 (16.6)
ȃMissing	6
**Node category†**	
ȃN0	398 (37.0)
ȃN1	380 (35.3)
ȃN2	297 (27.6)
ȃMissing	2
**Stage of disease at diagnosis**	
ȃI	25 (2.3)
ȃII	102 (9.5)
ȃIII	185 (17.2)
ȃIV	765 (71.0)
**Differentiation grade of colorectal cancer**	
ȃLow	15 (1.5)
ȃIntermediate	916 (92.0)
ȃHigh	65 (6.5)
ȃMissing	81
**Synchronous metastases**	797 (74.0)
**No. of liver metastases (n)**	
ȃMedian (i.q.r.)	2 (1–4)
ȃMissing	41
**Size of largest liver metastasis (mm)**	
ȃMedian (i.q.r.)	24 (16–36)
ȃMissing	83
**CEA (μg/l)**	
ȃMedian (i.q.r.)	9.0 (3.3–36.0)
ȃMissing	225
**Type of surgery**	
ȃLocal ablative therapy only	107 (9.9)
ȃWedge/segmental resection only	594 (55.2)
ȃMinor resection and local ablative therapy	178 (16.5)
ȃHemihepatectomy with or without ablation/wedge	198 (18.4)
**Resection margin status**	
ȃR0	841 (78.1)
ȃR1	141 (13.1)
ȃUnknown (tumour ablated)	95 (8.8)
**Perioperative systemic therapy**	
ȃNeoadjuvant only	322 (29.9)
ȃAdjuvant only	51 (4.7)
ȃPerioperative	54 (5.0)
ȃNone	650 (60.4)
**Tumour mutational status**	
ȃ*RAS*/*BRAF^V600E^* wild type	330 (47.1)
ȃ*BRAF^V600E^* mutation	19 (2.7)
ȃ*RAS* mutation	352 (50.2)
ȃMissing (*RAS* and/or *BRAF* status)	376
**MMR status**	
ȃMMR-deficient	15 (2.3)
ȃMMR-proficient	632 (97.7)
ȃMissing	430

*Values are *n* (%) unless indicated otherwise. †Of primary tumour. CEA, carcinoembryonic antigen; MMR, mismatch repair. Values are n (%) unless otherwise indicated.

### Patient outcomes after local treatment of CRLMs

During a median follow-up of 35 months, 807 recurrences were observed (73.0 per cent) and 249 patients died (23.1 per cent). Median OS was 51.3 (95 per cent c.i. 49.3 to not reached) months and RFS was 10.1 (9.5 to 10.9) months (*Fig. S1*). The site of first recurrence was liver-only in 332 patients (43.3 per cent) and extrahepatic (with or without intrahepatic metastases) in 399 (52.2 per cent) (*Table S2*). Median EHRFS was 20.4 (18.8 to 23.4) months. In the cohort, there were 557 EHR events (51.7 per cent) within 12 months, of which 194 (18.0 per cent) occurred within 6 months. Notably, 45 patients (23.2 per cent) with early EHR had undergone major liver surgery (hemihepatectomy), of which 23 (11.9 per cent of all patients) had two-stage resection, whereas only 15 patients (7.7 per cent) had local ablation therapy only.

The first EHR was a multisite EHR in 127 patients (26.6 per cent). The site of first EHR was most frequently the lungs and lymph nodes in 213 (44.6 per cent) and 55 (11.5 per cent) respectively, whereas the brain was affected in 6 patients (1.3 per cent). The site of first EHR correlated significantly with postrecurrence survival (*P* < 0.001); the shortest postrecurrence survival was in patients with brain metastases and longest in patients with EHR in the lymph nodes, within the abdomen or lungs (*Fig. S2*).

### Prognostic relevance of 6-month extrahepatic recurrence

Some 982 patients who survived until the landmark time (6 months after local treatment of CRLMs) were included in the landmark analysis to compare survival outcomes according to type of recurrence. Of those alive at 6 months after local treatment, 726 (73.9 per cent) had no recurrence, 123 (12.5 per cent) developed liver-only recurrence, and 133 (13.5 per cent) had EHR (including 100 patients with extrahepatic and intrahepatic recurrence). Median OS from the landmark time was 19.5 (95 per cent c.i. 15.6 to 23.0) months for patients with 6-month EHR after CRLM treatment (*Fig. S3*), 30.7 (29.0 to not reached) months for those with liver-only recurrence, and not reached (45.3 months to not reached) for patients without a recurrence.

### Prognostic value of tumour mutational status and sidedness of primary tumour

The prognostic value of tumour mutational status and sidedness of the primary tumour was first explored using univariable analysis for OS, EHRFS, and RFS (*Fig. S4* and *Table 2*). Median EHRFS for patients with *BRAF^V600E^*-mutated, *RAS*-mutated, and *RAS/BRAF^V600E^* wild-type tumours was 11.4 (95 per cent c.i. 5.8 to not reached), 18.5 (14.3 to 20.9), and 28.2 (22.2 to 33.9) months respectively (*P* < 0.005). The EHRFS for patients with right-sided, rectal, and left-sided tumours was 18.5 (13.3 to 32.0), 18.6 (14.5 to 23.8), and 23.0 (20.4 to 32.3) months respectively (*P* 0.039).

**Table 2:** Specifications of prediction model for extrahepatic recurrence-free survival

	n*	Univariable model		Full multivariable model		Selection model		
		HR	*P*	HR	*P*‡	HR	*P*‡	Shrunk HR
**Neoadjuvant treatment**					0.371			
ȃNot received	755	1.00 (reference)		1.00 (reference)		–		
ȃReceived	322	1.30 (1.05, 1.62)	0.018	0.89 (0.69, 1.15)		–		
**Tumour location**					0.074		0.080	
ȃRight colon	256	1.00 (reference)		1.00 (reference)		1.00 (reference)		
ȃLeft colon	460	0.73 (0.56, 0.95)	0.020	0.94 (0.71, 1.25)		0.94 (0.71, 1.25)		0.95
ȃRectum	361	0.96 (0.74, 1.26)	0.786	1.24 (0.94, 1.65)		1.24 (0.93, 1.64)		1.20
**Tumour category†**					0.007		0.010	
ȃT1, T2	153	1.00 (reference)		1.00 (reference)		1.00 (reference)		
ȃT3	744	1.29 (0.92, 1.80)	0.146	1.23 (0.87, 1.74)		1.22 (0.87, 1.72)		1.19
ȃT4	180	1.96 (1.33, 2.88)	<0.005	1.77 (1.18, 2.65)		1.72 (1.16, 2.56)		1.60
**Node category†**					<0.005		<0.005	
ȃN0	399	1.00 (reference)		1.00 (reference)		1.00 (reference)		
ȃN1	380	1.14 (0.88, 1.48)	0.305	1.21 (0.93, 1.58)		1.22 (0.94, 1.59)		1.19
ȃN2	298	1.77 (1.37, 2.28)	<0.005	1.64 (1.26, 2.13)		1.66 (1.28, 2.15)		1.55
**Tumour mutational status**					<0.005		<0.005	
ȃ*RAS/BRAF* wild type	505	1.00 (reference)		1.00 (reference)		1.00 (reference)		
ȃ*BRAF* mutation	44	2.08 (1.20, 3.59)	<0.010	2.16 (1.22, 3.82)		2.13 (1.21, 3.76)		1.92
ȃ*RAS* mutation	528	1.48 (1.16, 1.88)	<0.005	1.64 (1.26, 2.13)		1.67 (1.28, 2.16)		1.55
No. of liver metastases		–§		–§	<0.005	–§	<0.005	
Size of largest liver metastasis		–§		–§	<0.005	–§	<0.005	
Preoperative CEA		–§		–§	0.264	–		
Disease-free interval		–§		–§	0.367	–		

Values in parentheses are 95% confidence intervals. *Pooled number of patients for each level over the imputed data sets. †Of primary tumour. Specifications for univariable and multivariable Cox regression analyses for all candidate predictors for extrahepatic recurrence-free survival within 12 months after local treatment of colorectal liver metastases are shown, including the full multivariable models, and pooled selection model (extrahepatic recurrence model). For the pooled selection model, the apparent model HRs and the overfitting-adjusted HRs (shrunken) are shown (β_adjusted_ = β_unadjusted_ × shrinkage factor obtained via bootstrapping during internal validation, where β represents regression coefficient). ‡Multivariable Wald D1 *P* values. §Continuous variables were modelled non-linearly using restricted cubic splines; HRs are shown in *Fig. S5*.

### Extrahepatic recurrence prediction model

Following AIC-informed backward selection, the model included six of nine candidate predictor variables: sidedness of the primary tumour, T category, N category, *RAS/BRAF^V600E^* mutational status, and number and size of liver metastases; preoperative systemic treatment, preoperative CEA level, and DFI were not informative enough. Model HR values are shown in *Table 2* (non-linear HR plots for continuous variables can be found in *Fig. S5*). In an exploratory analysis including an interaction term between *RAS* mutational status and preoperative systemic treatment, the model fit did not significantly improve (*P* = 0.194, Wald’s D1 test).

### Performance and validation of model

**Fig. 1:**
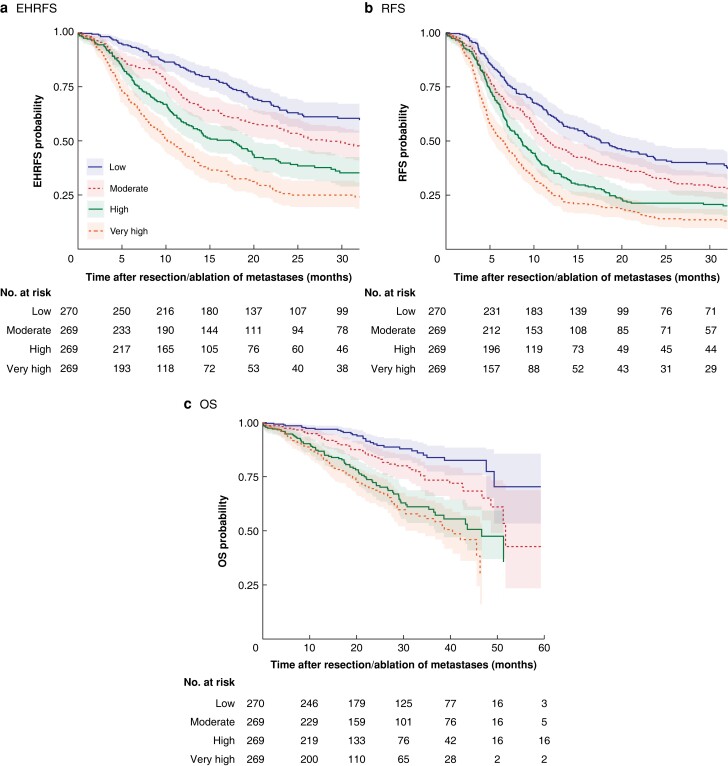
Kaplan–Meier curves for quartiles of predicted extrahepatic recurrence risk for three survival endpoints **a** Extrahepatic recurrence (EHR)-free survival (EHRFS), **b** recurrence-free survival (RFS), and **c** overall survival (OS). Predicted EHR risk includes EHR or death as an event for EHRFS. **a** Median EHRFS not reached, 28.2, 17.5, and 10.2 months for low-, moderate-, high-, and very high-risk quartiles repectively (*P* < 0.005); **b** median RFS 17.6, 11.5, 8.6, and 6.5 months respectively (*P* < 0.005); **c** median OS not reached, 51.7, 46.7, and 40.4 months respectively (*P* < 0.005) (log rank test). The survival probabilities were pooled over the imputed data sets after complementary log-log transformation.

EHRFS, RFS, and OS differed according to quartiles of predicted EHR risk (*Fig. 1*). Six-month EHR rates in the low-, intermediate-, high-, and very high-risk patient groups were 6.5 (95 per cent c.i. 3.9 to 9.9), 15.0 (11.0 to 19.6), 20.3 (15.7 to 25.4), and 32.0 (26.4 to 37.7) per cent respectively. Likewise, the model showed good discrimination for RFS and OS.

**Fig. 2:**
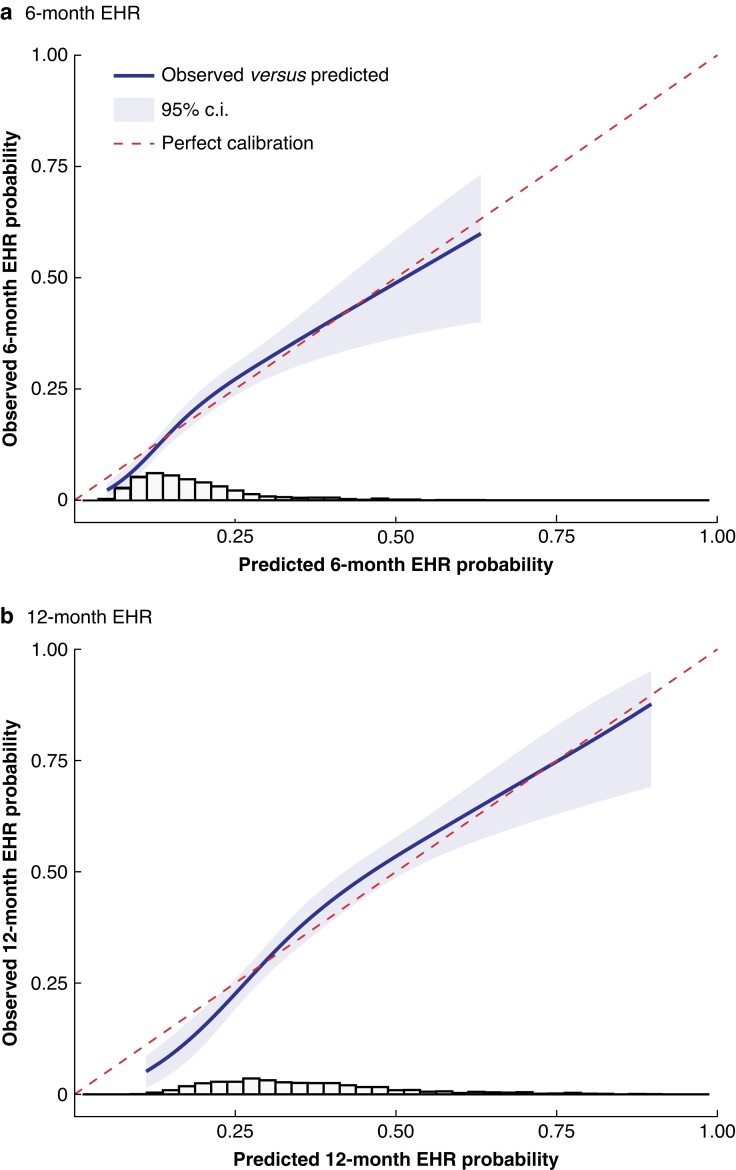
Calibration plots for predicted *versus* observed 6- and 12-month extrahepatic recurrence Predicted *versus* observed **a** 6-month and **b** 12-month extrahepatic recurrence (EHR, which includes EHR or death as events for EHR-free survival) probabilities. The histogram shows the distribution of predicted EHR probabilities. The integrated calibration index was 0.015 (6-month EHR) and 0.028 (12-month EHR). The median absolute difference was 0.017 (6 months) and 0.030 (12 months), with a maximum absolute difference of 0.03 (6 months) and 0.06 (12 months).

The performance of the prediction model was further assessed by calibration and discrimination. The estimated and observed risks for EHR or death were well calibrated (*Fig. 2*). The observed to expected ratio was 1.015 (95 per cent c.i. 0.911 to 1.120). For discrimination, Harrell’s C-index through 6 and 12 months was 0.663 (95 per cent c.i. 0.624 to 0.702) and 0.661 (0.632 to 0.689) respectively, and similar for Uno’s C-index. The 6- and 12-month areas under the time-dependent ROC curves were 0.668 (95 per cent c.i. 0.626 to 0.709) and 0.671 (0.636 to 0.707) respectively (*Fig. S6*). The shrinkage factor obtained through internal validation was 0.86; shrunken HRs are shown in *Table 2*. The shrunken model yielded overoptimism-corrected 6-month risks for EHR or death of between 5.9 and 56.0 per cent (i.q.r. 12.9–22.0 per cent). The optimism-adjusted Harrell’s C-index through 6 and 12 months was 0.643 (0.605 to 0.682) and 0.641 (0.612 to 0.669). Full model specifications are shown in *Appendix S1*.

The model was further validated for generalizability by internal–external cross-validation using three geographical regions, which indicated that models developed on the other regions showed adequate performance in each excluded geographical region (*Fig. S7*).

### Decision curve analysis for net benefit when using model-guided CRLM treatment decisions

**Fig. 3:**
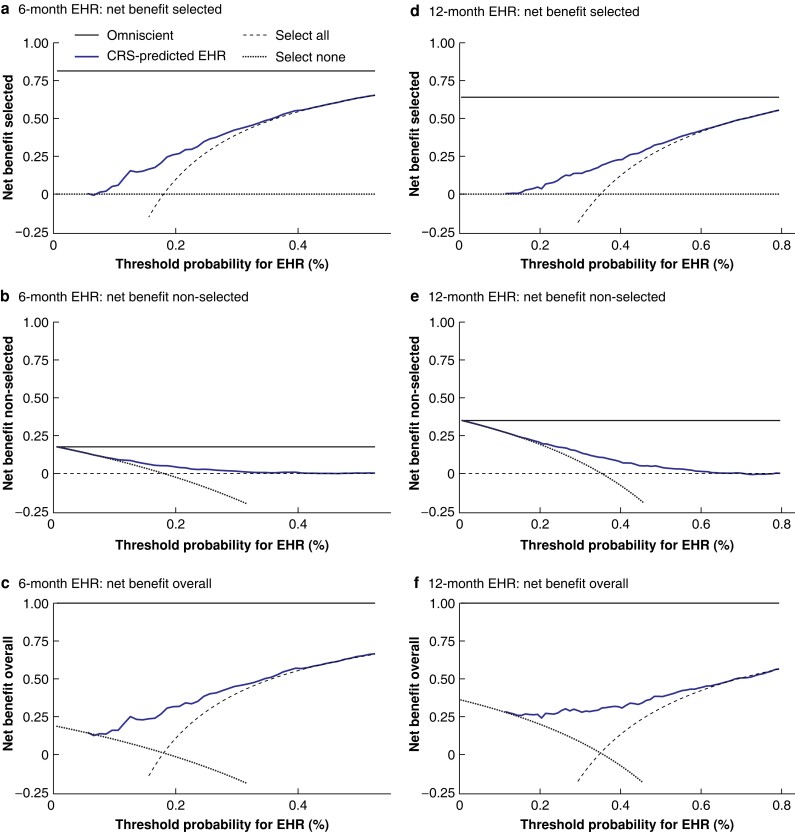
Decision curve analysis plots Plots indicate the net benefit obtained for a given threshold value for **a–c** 6-month and **d–f** 12-month extrahepatic recurrence (EHR) probability, which includes EHR or death as an EHR-free survival (EHRFS) event. The net benefit was compared across three situations: non-informed decision-making (selecting all patients or no patients (dashed and dotted lines respectively)) and for informed decision-making by selecting patients for local treatment of colorectal liver metastases (CRLMs) according to the clinical risk score's (CRS) predicted EHRFS probability (blue continuous line). For comparison, the horizontal black continuous line represents an omniscient model (all-knowing model). **a**,**d** Net benefit of local treatment for CRLMs (selected patients) is determined using the true-positives (patients with predicted EHRFS probability (p_EHRFS_) above the threshold value and not having had an EHR) *versus* false-positives (p_EHRFS_ above threshold and the patient did have an EHR) for a range of threshold values (0–1), with the benefit of false-positives weighted relative to the threshold value. For consistency, the net benefit is shown for a range of thresholds for EHR (EHR probability = 1 – EHRFS probability). **b**,**e**. Net benefit of no local treatment for CRLMs (non-selected patients) is determined using the true-negatives (patients with p_EHRFS_ below threshold and having an EHR) *versus* false-negatives (patients with p_EHRFS_ below threshold and not having had an EHR) for a range of threshold values (0–1), with the benefit of false-negatives weighted relative to the threshold value. **c**,**f**. Overall net benefit is the sum of the net benefit for the selected and non-selected patients.

The potential net benefit of the model for clinical decision-making regarding local treatment of CRLMs was examined through decision curve analysis. EHR model-guided treatment of CRLMs (compared with non-informed decision-making by treating all or no patients) resulted in net benefit for patients for 6-month EHR risk thresholds of 0–40 per cent and 12-month EHR risk thresholds of 0–60 per cent (*Fig. 3*).

## Discussion

In this study, a prediction model was developed for early EHR in a nationwide, population-based cohort of patients who had local treatment of CRLMs. The model incorporated tumour *RAS/BRAF^V600E^* mutational status and sidedness of primary tumour alongside traditional prognostic factors. Early EHR after local CRLM treatment is of major clinical importance and can be predicted from routine clinical information. The EHR prediction model developed here discriminates between patients based on EHR rates, reflected in differing EHRFS, RFS, and OS. The EHR prediction model’s expected generalizability is good.

Prediction models are increasingly being used, and can facilitate shared risk-informed decision-making for interventions, manage patient expectations, or select patients for inclusion in trials. However, clinical application of prediction models for local CRLM treatment is hampered by lack of generalizability, loss of predictive performance by simplification of models, and low clinical utility^37^. Published models were developed to predict RFS and OS. With increasing possibilities for repeated resections of CRLM recurrences with favourable survival outcomes^16,17^, RFS and OS prediction models become less relevant. The present study confirmed that about half of patients have a liver-limited first recurrence and experience long-term survival. Although RFS and OS are meaningful outcomes to manage expectations, EHRFS as outcome may guide clinical decisions for patients with CRLMs.

Local CRLM treatment should ideally be avoided in patients who experience early EHR (18.0 per cent of patients). These patients evidently have systemic disease, a poor prognosis, and are often not eligible for repeated local treatment^18–23^. The poor OS demonstrated in patients with early EHR (19.5 months in landmark analysis) is comparable to the expected OS of patients with metastatic colorectal cancer undergoing palliative systemic treatment^42^. Patients at high risk of early EHR are unnecessarily exposed to potential perioperative risks and may be harmed by delaying palliative systemic treatment, especially as a large proportion of the high-risk patients underwent major liver surgery as they had more extensive disease. The EHR prediction model can be used to confirm that local treatment should be pursued in low-risk patients. However, it is currently difficult for the EHR prediction model to identify patients with a sufficiently high predicted risk that would justify avoiding local CRLM treatment. The EHR prediction model may aid clinical decision-making by identifying moderate–high-risk patients for early EHR who may benefit from perioperative systemic treatment. A treatment strategy for these patients may be to initiate systemic treatment and, upon sustained response, carry out local treatment of CRLMs. Once externally validated, the EHR model will lend itself well for studies examining the optimal treatment by stratifying patients who are at moderate–high risk of early EHR.

The strength of the study is a nationwide cohort of patients encompassing 39 academic, teaching, and regional hospitals. The cohort had minimal loss to follow-up (below 1.0 per cent). Furthermore, the EHR prediction model included *RAS* and *BRAF^V600E^* mutational status, important prognostic factors. Only three previous prediction models included *RAS* and *BRAF* mutation status^13–15^, potentially owing to the low prevalence of *BRAF* mutations in patients with local treatment of CRLMs (approximately 2 per cent)^13^. In contrast to previous studies^28,43^, there was no interaction between neoadjuvant treatment status and *RAS* mutational status here.

Limitations include a selected population based on primary tumour diagnosis in 2015 and 2016, with subsequent local treatment of CRLMs until January 2019 (no DFI beyond 4 years). The prediction model could not robustly specify site of recurrence, which may be relevant especially for patients with lung-only recurrences who can experience long-term survival after local treatment^44,45^. It was not possible to validate the prediction model externally beyond internal–external cross-validation. The full EHR prediction model specifications have been provided to facilitate external validation in other patient cohorts.

The performance of the model could be improved further by including additional promising features that may better identify high-risk patients^15^. Examples include distinct histopathological growth patterns, the Immunoscore (based on T cell infiltration), a six-gene panel, and liquid biopsies (detecting circulating tumour DNA)^46–49^. Incorporating these features into an updated prediction model for local CRLM treatment may help identify patients at sufficiently high risk for early EHR to optimize the treatment strategy for such patients.

## Supplementary Material

znac461_Supplementary_Data

## Data Availability

The data sets generated during and analysed during this study are not publicly available owing to NCR regulations, but are available from the corresponding author or NCR on reasonable request.
