# Supplementary material for: Predicting early extrahepatic recurrence after local treatment of colorectal liver metastases
Source: Br J Surg. 2023 Jan 19;110(3):362–71. doi: 10.1093/bjs/znac461 (PMC10364507; doi:10.1093/bjs/znac461)
Supplement: znac461_Supplementary_Data [file znac461_supplementary_data.docx]

**Predicting early extrahepatic recurrence after local treatment of colorectal liver metastases**

G. Emerens Wensink MD^1^*, Karen Bolhuis MD^2,3^*, Marloes A.G. Elferink PhD^4^, Remond J.A. Fijneman PhD^5^, Onno Kranenburg PhD^6,7^, Inne H.M. Borel Rinkes MD PhD^6^, Miriam Koopman MD PhD^1^, Rutger-Jan Swijnenburg MD PhD^8^, Geraldine R. Vink MD PhD^1,4^, Jeroen Hagendoorn MD PhD^6^, Cornelis J.A. Punt MD PhD^9^, Jeanine M.L. Roodhart MD PhD^1^**, Sjoerd G. Elias MD PhD^9^**

^1^University Medical Center Utrecht, Department of Medical Oncology, Utrecht University, Utrecht, the Netherlands

^2^ Amsterdam UMC location University of Amsterdam, Department of Medical Oncology, Amsterdam, the Netherlands

^3^Netherlands Cancer Institute, Department of Gastrointestinal Oncology, Amsterdam, Netherlands

^4^The Netherlands Comprehensive Cancer Organisation (IKNL), Department of Research and Development, Utrecht, the Netherlands

^5^The Netherlands Cancer Institute, Department of Pathology, Amsterdam, the Netherlands

^6^University Medical Center Utrecht, Department of Surgery, Utrecht University, Utrecht, the Netherlands

^7^Utrecht Platform for Organoid Technology, University Medical Center Utrecht, Utrecht University, Utrecht, the Netherlands

^8^Amsterdam UMC location University of Amsterdam, Department of Surgery, Amsterdam, the Netherlands

^9^Julius Center for Health Sciences and Primary Care, Department of Epidemiology, University Medical Center Utrecht, Utrecht University, Utrecht, the Netherlands.

* Shared first author, **shared last author

**Corresponding author.**

Dr. Sjoerd Elias

Julius Center for Health Sciences and Primary Care, Department of Epidemiology

University Medical Center Utrecht, Utrecht University,

Universiteitsweg 100, 3584 CX, Utrecht, The Netherlands

**Supplementary Materials - Index**

| **Supplementary Methods** |  |
| --- | --- |
| Supplementary Methods | *pag. 3* |
| **Supplementary Appendixes** |  |
| **Appendix S1.** Formulas to predict extrahepatic recurrence risk at 6 and 12 months following local treatment of CRLM | *pag. 6* |
| **Supplementary Figures and Tables** |  |
| **Table S1.** Assumptions regarding systemic treatment and survival outcomes | *pag. 8* |
| **Figure S1.** Kaplan-Meier analysis showing RFS, EHRFS and OS with 95% confidence intervals of the total cohort | *pag. 10* |
| **Table S2.** Detailed information about first recurrence and first extrahepatic recurrence | *pag. 11* |
| **Figure S2**. Post-recurrence overall survival of patients according to site of extrahepatic recurrence | *pag. 12* |
| **Figure S3.** Landmark analysis at six months showing Kaplan-Meier curves of patients with extrahepatic, intrahepatic-only and no recurrences | *pag. 13* |
| **Figure S4.** Kaplan-Meier curves describing post-resection overall survival, recurrence-free survival and extrahepatic recurrence-free survival in the total cohort according to location of primary tumour and *RAS/BRAF* mutation status. | *pag. 14* |
| **Figure S5.** Hazard ratio for EHRFS for continuous variables modeled using restricted cubic splines | *pag. 16* |
| **Figure S6.** Time-dependent ROC curve for the EHRFS model indicating the true positive and false positives 6 and 12 months after local treatment for CRLM | *pag. 18* |
| **Figure S7.** Internal-external cross-validation results | *pag. 19* |
| **References** | *pag. 20* |

**Supplementary Methods**

*Early EHR as primary endpoint for the prediction model*

By consensus of experts in the field (JR, RJS, KB, EW, JH, MK, CJAP), early EHR (within six months, conform previous publications^1,2^) was defined as the clinically relevant primary endpoint of the model, due to the poor prognosis in patients with early EHR and lower chance of repeat local treatment, in contrast to patients with liver-only recurrences. Thus, the added value of local treatment of CRLM may not be justified in patients with a rapid EHR after local treatment of CRLM by consensus of experts in the field.

*Statistical analysis*

We used standard descriptive statistics to describe baseline characteristics of the study population, including medians and interquartile intervals (IQI) for continuous variables, and frequency and percentages for categorical variables. Follow-up data and patient outcomes were described using (reverse) Kaplan Meier approaches. We assessed the prognostic impact of our primary endpoint occurrence of EHR ≤6 months after CRLM treatment using landmark analysis - which prevents immortal time bias - at six months after CRLM treatment and comparing the subsequent survival outcomes of three groups based on site of recurrence ≤6 months: no recurrence, intrahepatic only and EHR (which includes patients with intra- and extrahepatic recurrences).

We applied the recommendations published by Riley *et al.*^3^, to determine the number and complexity of the candidate predictors (together amounting to the number of coefficients) to be evaluated in our prediction model. We used the C-index for the Comprehensive Evaluation of Relapse Risk (CERR) score^4^ as the anticipated minimum C-index for our model, since it most closely represents our primary end-point. With an expected C-index of ≥0.695, and the observed EHR event rate within 12 months in our cohort, we had sufficient data to model 17 coefficients and fulfill the 3 criteria set by Riley *et al.*^3^.

Predictors were selected by assessment of a multidisciplinary team based on factors used in previous prediction models^5–8^ and newly recognized prognostic factors^9,10^. Candidate predictors were blindly selected, prior to having analyzed the data. Nine candidate predictors were selected for model development, including 4 continuous variables that we aimed to model using three-knot restricted cubic splines (rcs) to allow for non-linearity (resulting in 17 coefficients): neoadjuvant systemic treatment, primary tumour location (left-sided (splenic flexure-rectosigmoid), right-sided (coecum-transverse colon), rectum), T-status (T1-2, T3, T4), N-status (N0, N1, N2), *RAS/BRAF* mutational status (*RAS/BRAF*-wildtype, *RAS*-mutant, *BRAF*-mutant), number of liver metastases (continuous), size of largest liver metastasis (continuous), pre-operative CEA (continuous) and DFI (continuous). Continuous variables were Winsorized at the 95^th^ percentile before analyses to decrease influential points. As certain candidate predictors had missing data, and merely removing patients with missing data leads to loss of information and potentially also to selection bias, we used multiple imputation using multivariate imputation by chained equations (MICE)^11^, assuming missingness at random.

The imputation model contained all above selected candidate predictor variables including rcs transformations to accommodate congeniality, and included sidedness of the primary tumour, age, T-status, N-status, *RAS/BRAF^V600E^* mutational status, number and size of liver metastases, serum CEA, DFI, systemic perioperative treatment type, R-status, tumour burden score (TBS^12^, passively imputed in the model as auxiliary variables, as well as the primary outcome (EHRFS within 12 months using a Nelson-Aalen estimator and event indicator). We generated 53 imputed datasets, based on the percentage of patients with at least one missing variable in the candidate predictor variables set. To enable internal-external cross-validation without information leakage, we imputed the data separately for each geographical region.

*Developing, validating and assessing performance of clinical risk score*

Following multiple imputation, a prediction model for EHRFS within 12 months after local treatment of CRLM (EHRFS model) was created using Cox regression, ignoring follow-up information beyond 12 months. This 12-month time-horizon was chosen to allow a sufficient number of events for robust model-development based on the criteria by Riley *et al.*^3^. The primary evaluation of resulting model's performance was early EHR (≤6 months). Because of the limited evaluated follow-up period we did not evaluate deviations from the proportionality assumption.

The primary prediction model was developed in the whole cohort, using a Cox proportional hazards model with Akaike Information Criterion (AIC)-based backward selection in each imputed dataset leading to a primary model only including predictors selected in ≥50% of imputed datasets, which was then refitted in each imputed dataset to obtain a pooled selection model using Rubin's rules (**Supplementary Methods Figure 1**). The only variable that was not subjected to the above was adjuvant systemic therapy, which was included in all models using an offset for expected therapeutic efficacy (i.e. this effect was not estimated from the data but was imposed on the model by the offset). For the expected adjuvant systemic treatment effect we used the pooled random effects hazard ratio (HR) from known randomized controlled trials in the literature^13,14^, resulting in a HR of 0.73.

**Supplementary Methods Figure 1.** Model development, internal validation and internal-external cross-validation for development of prediction model


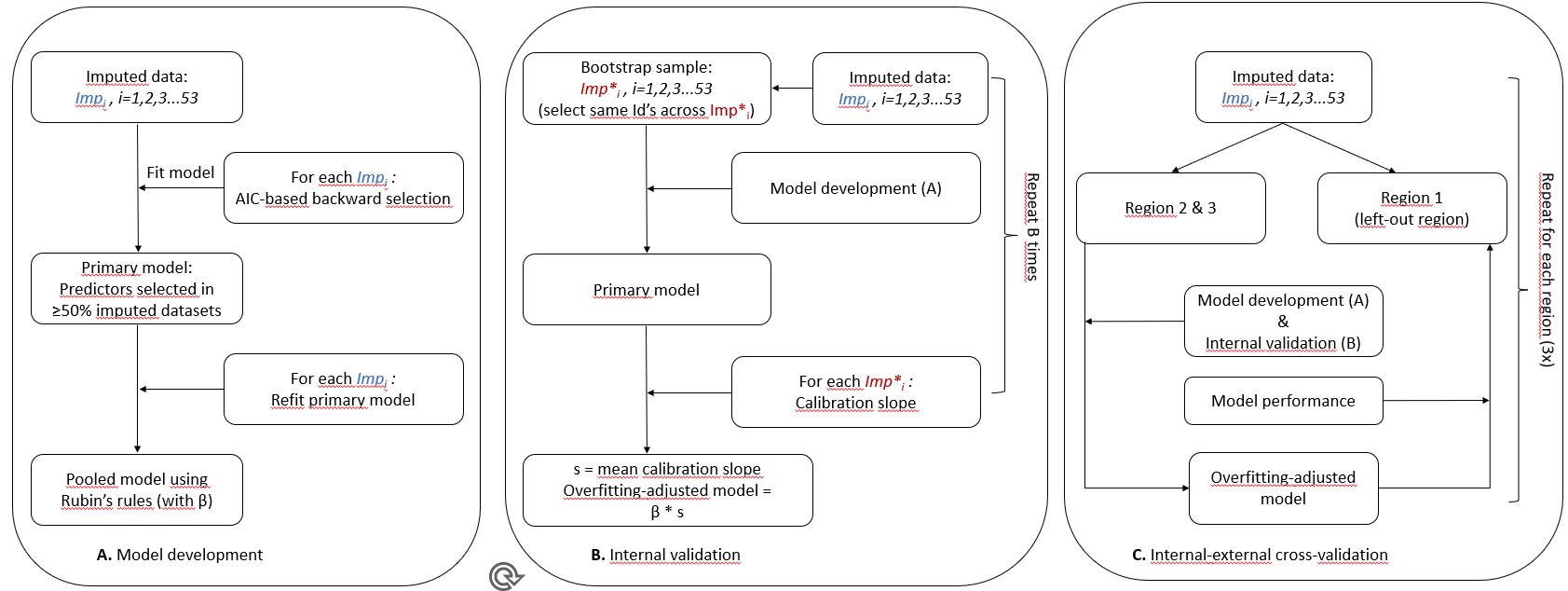


Legend. A flowchart illustrating the steps in model development (A), internal validation (B) and internal-external cross-validation (C), adapted from ^15,16^. The EHRFS model was developed by performing AIC-based backward selection of all candidate predictors in each imputed dataset. The selection model included variables which were selected in ≥50% of the imputed datasets. The EHRFS model regression coefficients were pooled using Rubin’s rules. Internal validation was performed using 500 bootstrap samples to determine the shrinkage factor and overfitting-adjusted model. The model development and internal validation was repeated for internal-external cross-validation, using geographical regions in the development and validation cohorts. The cross-validation was repeated for all regions, resulting in three performance measures.

*Abbreviations:* AIC (Akaike Information Criteria), B (number of bootstrap samples), β (regression coefficient), *Imp** (bootstrap object of an imputed dataset), *Imp* (imputed dataset), s (shrinkage factor)

Model performance was assessed using calibration plots for 6 and 12 month EHR risk, discrimination (Harrell’s C-index, Uno’s C-index through 6 and 12 months), time-dependent receiver operator characteristic (ROC) curve, Nagelkerke’s R^2^ and decision-curve analysis. Each measure was determined for each imputed dataset separately and pooled using Rubin’s rules (**Supplementary Methods Figure 1**). Model-predicted and Kaplan-Meier observed survival estimates (and 95% CI boundaries) were pooled after complementary log-log transformation, and Nagelkerke’s R^2^ was pooled after Fisher z-transformation. Decision curve analysis was used to assess the net benefit associated with CRLM treatment decisions based on a given threshold value for 6-month or 12-month EHRFS probability^17^. To visualize the potential relevance of the developed model we used Kaplan-Meier curves for EHRFS, RFS and OS, categorizing patients based on quartiles of (across-imputation dataset pooled) predicted EHR risk. The reported performance measures were based on predicted EHR-risks which included the effect of adjuvant treatment (if given).

To quantify the overoptimism of the model regarding predicted risks and discriminative ability, we used internal validation by 500-fold bootstrap resampling, repeating all model-development steps in each bootstrap sample and testing the performance of the resulting models from each bootstrap in the original data. We derived a uniform shrinkage factor from internal validation that we applied to the apparent pooled regression coefficients of the primary model as fitted in the original data to create an overoptimism-corrected model (the offset for adjuvant systemic therapy was not shrunk). This overoptimism-corrected model yields predicted EHR probabilities that will agree more with actual risk in new patients. We similarly obtained overoptimism-corrected C-indexes that likely better reflect the actual discrimination of our model in new patients.

We used internal-external cross-validation to evaluate the generalizability of the model (**Supplementary Methods Figure 1**). The data were split in three geographic regions and all above described modeling steps including internal validation were repeated in two of three regions, after which the performance of the overfitting-adjusted model was evaluated in the left-out geographical region (C-index, calibration slope and intercept). Each geographic region was left-out of model development once, resulting in three estimates of external validation.

As an exploratory additional analysis, we tested whether the prognostic value of *RAS* mutation for EHRFS depended on the administration of preoperative systemic treatment which was reported^18,19^, by using a D1 test between a multivariable model without and with a *RAS**preoperative systemic treatment interaction term. To avoid bias, two separate multiple imputation models were created for patients based on preoperative systemic treatment status solely for the exploratory analysis.

**Supplementary Appendixes**

**Appendix S1.** Formulas to predict extrahepatic recurrence risk at 6 and 12 months following local treatment of CRLM

This appendix is added to the manuscript in line with the TRIPOD recommendations.

The distribution of patients with 6-month (A) and 12-month (B) predicted extrahepatic recurrence risk according to the apparent model is illustrated below. The top light grey values include an offset for patients who received adjuvant systemic treatment, whereas the lower dark grey values indicate the predicted probabilities if patients had not received adjuvant systemic treatment.


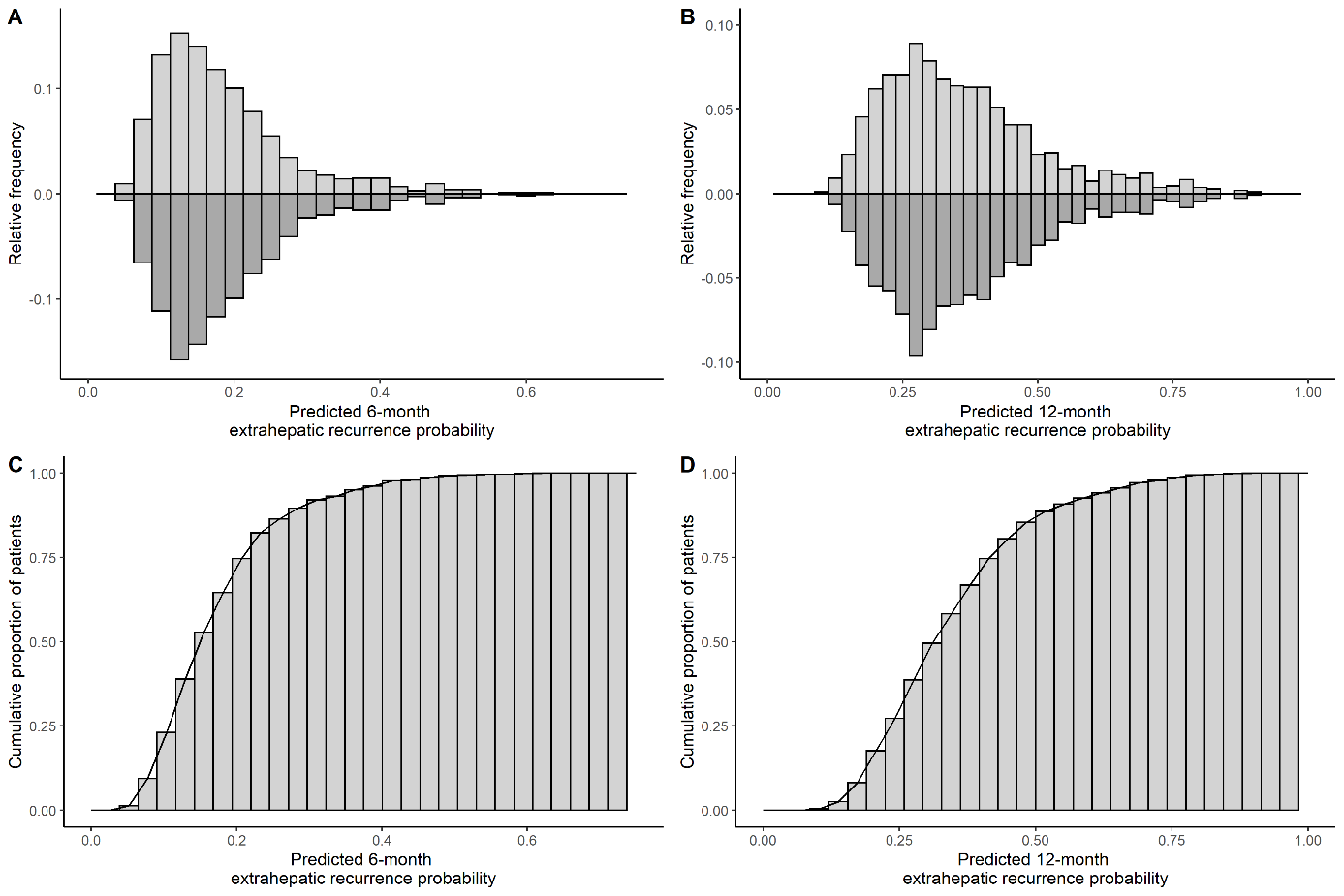


Adjuvant systemic therapy was included in the model using an offset for expected therapeutic efficacy (i.e. this effect was not estimated from the data but was imposed on the model by the offset) based on the published hazard ratio, resulting in a HR of 0.73.

**A.** As observed in the analyzed dataset.

Baseline cumulative hazard at 6-months:

*H*_0_(*t_6 months_*) = 0.0416

Baseline cumulative hazard at 12-months:

*H*_0_(*t_12 months_*) = 0.0947

Prognostic index (PI; linear predictor):

PI = - 0.061*X _Left-sided primary tumour location_ + 0.212*X _Rectum primary tumour location_

+ 0.198*X _T3 upon diagnosis_ + 0.543*X _T4_

+ 0.200*X _N1 upon diagnosis_ + 0.507*X _N2_

+ 0.758*X *_BRAF_*_-mutant_ + 0.510*X *_RAS_*_-mutant_

+ *f* (Number of liver metastases)

+ *f* (Size of largest liver metastasis)

- 0.314*X _Adjuvant systemic treatment_

Where number of liver metastases and size of largest liver metastasis are described with a restricted cubic spline function:

*f* (Number of liver metastases) = + 0.101*Number of liver metastases

+ 0.00194*max(Number of liver metastases -1,0)^3^

- 0.0023*max(Number of liver metastases-2,0)^3^

+ 0.000389*max(Number of liver metastases-7,0)^3^

*f* (Size of largest liver metastasis) = + 0.017*Size of largest liver metastasis

- 3.332*10^-6^*max(Size of largest liver metastasis-11,0)^3^

+ 4.535*10^-6^*max(Size of largest liver metastasis-24,0)^3^

- 1.203*10^-6^*max(Size of largest liver metastasis-60,0)^3^

The absolute predicted extrahepatic recurrence risk at time *t*:

Risk = 1-exp(-(exp(PI)**H*_0_(*t*)))

Example:

The 6-month predicted extrahepatic recurrence risk for a patient with a right-sided primary tumour location, T3 tumour stage and N1 nodal stage upon diagnosis, a *RAS-*mutation and with 1 liver metastasis upon diagnosis of CRLM (size 23 mm), who received adjuvant systemic treatment:

*H*_0_(*t*=6) = 0.0416

PI = - 0.061*0 + 0.212*0 + 0.198*1 + 0.543*0 + 0.200*1 + 0.507*0 + 0.758*0 + 0.510*1

+ 0.101*1 + 0.00194*max(1 -1,0)^3^ - 0.0023*max(1-2,0)^3^ + 0.000389*max(1-7,0)^3^

+ 0.017*23 - 3.332*10^-6^*max(23-11,0)^3^ + 4.535*10^-6^*max(23-24,0)^3^ - 1.203*10^-6^*max(23-60,0)^3^

- 0.314*1

= 1.08

Risk = 1-exp(-(exp(1.08)*0.0416))

= 0.115 = 11.5%

**B.** Following correction for overoptimism.

Correction for overfitting was by 500-fold bootstrap resampling as internal validation. Since adjuvant systemic treatment was modelled using an offset term, its coefficient does not undergo shrinkage.

Baseline cumulative hazard at 6-months:

*H*_0_(*t_6 months_*) = 0.1882

Baseline cumulative hazard at 12-months:

*H*_0_(*t_12 months_*) = 0.4249

Prognostic index (PI; linear predictor):

PI = - 0.053*X _Left-sided primary tumour location_ + 0.183*X _Rectum primary tumour location_

+ 0.171*X _T3 upon diagnosis_ + 0.469*X _T4_

+ 0.172*X _N1 upon diagnosis_ + 0.438*X _N2_

+ 0.654*X *_BRAF_*_-mutant_ + 0.440*X *_RAS_*_-mutant_

+ *f* (Number of liver metastases)

+ *f* (Size of largest liver metastasis)

- 0.314*X _Adjuvant systemic treatment_

Where number of liver metastases and size of largest liver metastasis are described with a restricted cubic spline function:

*f* (Number of liver metastases) = + 0.087*Number of liver metastases

+ 0.00167*max(Number of liver metastases -1,0)^3^

- 0.0020*max(Number of liver metastases-2,0)^3^

+ 0.000333*max(Number of liver metastases-7,0)^3^

*f* (Size of largest liver metastasis) = + 0.015*Size of largest liver metastasis

- 2.915*10^-6^*max(Size of largest liver metastasis-11,0)^3^

+ 3.968*10^-6^*max(Size of largest liver metastasis-24,0)^3^

- 1.053*10^-6^*max(Size of largest liver metastasis-60,0)^3^

The absolute predicted extrahepatic recurrence risk at time *t*:

Risk = 1-exp(-(exp(PI)**H*_0_(*t*)))

**Supplementary Figures and Tables**

**Table S1.** Assumptions regarding systemic treatment and survival outcomes

| Assumptions regarding progression of disease and survival: |
| --- |
| Date of new episode is considered as time of progression. |
| When disease progression is documented < 14 days of liver resection we assume this was part of the liver resection and first new episode is considered as time of progression. |
| Recurrence-free survival (RFS) is calculated from date of first liver procedure to date of progression. In case of 2-stage resection, RFS is calculated from last liver procedure. |
| RFS is calculated from date of first liver procedure to date of progression. In case of 2-stage resection, RFS is calculated from last liver procedure. |
| If no recurrence is registered:  If end of follow-up is registered and reason end of follow-up is: death, then date of death is registered as event of RFS;  If end of follow-up is registered and reason end of follow-up is other than death then RFS is censored on date of end of follow up;  If no date of end of follow-up is registered then RFS is censored on date of last visit;  If none of these dates are registered then RFS is documented as missing. |
| Extrahepatic recurrence-free survival (EHRFS) is calculated from date of first liver procedure to date of progression. In case of 2-stage resection, EHRFS is calculated from last liver procedure. |
| If no extrahepatic recurrence is registered:  If end of follow-up is registered and reason end of follow up is: death, then date of death is registered as event of EHRFS;  If end of follow-up is registered and reason end of follow up is other than death then EHRFS is censored on date of end of follow up;  If no date of end of follow-up is registered then EHRFS is censored on date of last visit;  If no last visit is registered but event for RFS is registered then EHRFS is censored on date of RFS;  If none of these dates are registered then EHRFS is documented as missing. |
| Extrahepatic disease was defined as presence of disease outside the liver or metastasectomy outside the liver. |
| Lymph node metastases registered as abdominal lymph nodes at time of first liver metastases were considered extrahepatic disease and as so classified as not-liver only disease. |
| Overall survival (OS) is calculated from date of diagnosis of metastatic disease. |
| Patients who did not die are censored on the date last known to be alive in the GBA (the municipal population registry). |
| OS after resection is calculated from date of first liver procedure. In case of 2-stage resection, OS is calculated from date of last liver procedure. |
| Primary tumour nodal stage was defined primarily on pathologic N-stage. When pN stage was missing, cN stage (radiological) was used. |
| If number of metastases was not given and code 77 was used (accounting for diffuse metastatic disease in the liver) then number of metastases was scored as 20. |
| If performance status was missing, this was scored as 0-1 because patients were considered physically good enough for resection. |
| Assumptions regarding systemic treatment regimens and strategies: |
| Systemic treatment includes both chemotherapy and/or targeted therapy. |
| A combination regimen is defined as all systemic agents starting within 4 weeks after start of the first agent and started before progression of disease. |
| If bevacizumab was started more than 4 weeks after the start of the first agent but before stop of this agent and before progression of disease, we assume bevacizumab was part of this combination regimen. |
| If a treatment line continues despite of progression, e.g., in case of reintroduction of the same or an equivalent regimen after a therapy break and detected progression, we regard this as continuation of the same treatment line. |
| If oxaliplatin only is registered, we assume this was part of a capecitabine and oxaliplatin (CAPOX) regimen of which capecitabine was not registered, so we add capecitabine. We assume this is due to a registration error, in which the administration of capecitabine has not been noticed by the data manager. |
| Systemic therapy was considered adjuvant systemic therapy for primary tumour when started < 12 weeks after resection of primary tumour and started before diagnosis of metastases in patients with metachronous disease. |
| Capecitabine monotherapy was considered radiosensitizer for primary tumour when started before primary tumour resection and before diagnosis of metastases and with notification to have received chemoradiotherapy. |
| Systemic therapy was considered pre-operative therapy (neo-adjuvant or induction) before liver resection when the therapy ended within 120 days before liver resection. Adjuvant therapy after resection of primary tumour or chemotherapy as radiosensitizer was excluded. |
| Systemic therapy was considered adjuvant therapy after liver resection when the therapy started within 120 days after liver resection. Chemotherapy as radiosensitizer was excluded. |
| Systemic therapy was considered peri-operative therapy of liver resection when the systemic therapy was given <120 days before and < 120 days after liver resection |
| When systemic therapy was given between two liver procedures before progression of disease, the first liver procedure was considered as staging procedure and systemic therapy was considered as pre-operative systemic therapy (neo-adjuvant or induction) for surgery 2 |
| Treatment strategies are categorized as follows:  Treatment regimens containing chemotherapy, without targeted therapy, subdivided in: monotherapy (1 chemotherapy agent), doublets (2 chemotherapy agents) and triplets (3 chemotherapy agents);  Treatment regimens containing targeted therapy with or without chemotherapy, subdivided in: bevacizumab-containing regimens, and anti-EGFR targeted therapy-containing regimens. |
| Systemic therapy regimens are categorized as follows:  Fluoropyrimidine monotherapy (e.g. 5-fluorouracil [5-FU], capecitabine);  Oxaliplatin-based doublet therapy (e.g. capecitabine + oxaliplatin (CAPOX), 5-FU/oxaliplatin [FOLFOX]);  Irinotecan-based doublet therapy (e.g. capecitabine + irinotecan (CAPIRI), 5-FU/irinotecan [FOLFIRI])  Triplet systemic therapy (5-fluorouracil [5-FU], oxaliplatin and irinotecan)  Targeted therapy (anti-EGFR therapy; cetuximab or panitumumab, and bevacizumab) |
| A treatment line is defined as systemic therapy (monotherapy or combination regimen) administered at the same time until suspension, regardless of reason for discontinuation. |
| Treatment is considered as next line if an agent of a new drug group is started that is not applied in the previous systemic treatment regimen. |
| If the same or an equivalent systemic treatment regimen is (re)started, this is considered continuation of the same treatment line, e.g., CAPOX to FOLFOX. |
| Assumptions regarding mutational status: |
| *RAS* and *BRAF* mutation are considered mutual exclusive, therefore patients with *RAS* mutations or *BRAF* mutations, were assumed to have *BRAF-*wildtype or *RAS-*wildtype status, retrospectively. |
| Assumptions regarding systemic treatment lines: |
| A treatment line is defined as systemic therapy (monotherapy or combination regimen) administered at the same time until suspension, regardless of reason for discontinuation. |
| Treatment is considered as next line if an agent of a new drug group is started that is not applied in the previous systemic treatment regimen. |
| If the same or an equivalent systemic treatment regimen is (re)started, this is considered continuation of the same treatment line, e.g. CAPOX to FOLFOX. |
| Assumptions regarding local treatment |
| Local treatments are categorized as follows: 1 stage (1 procedure); 2-stage (2 procedures < 120 days apart) |
| R-status:  when two stage procedure and first procedure was R2 resection and second procedure was R1/R0 resection then 2-stage resection considered as R-status of last procedure.  when 2-stage resection and one procedure was R1 resection and other local treatment was R0 resection then considered as R1 resection. |

*Abbreviations:* 5-FU (5-flouruoracil), CAPIRI (capecitabine + irinotecan), CAPOX (capecitabine + oxaliplatin), cN (radiological nodal-stage), EGFR (epidermal growth factor receptor), EHRFS (extrahepatic recurrence-free survival), FOLFIRI (5-FU + irinotecan), FOLFOX (5-FU + oxaliplatin), GBA (the municipal population registry), OS (overall survival), pN (pathological nodal-stage), RFS (recurrence-free survival).

**Figure S1.** Kaplan-Meier analysis showing RFS, EHRFS and OS curves of the total cohort.


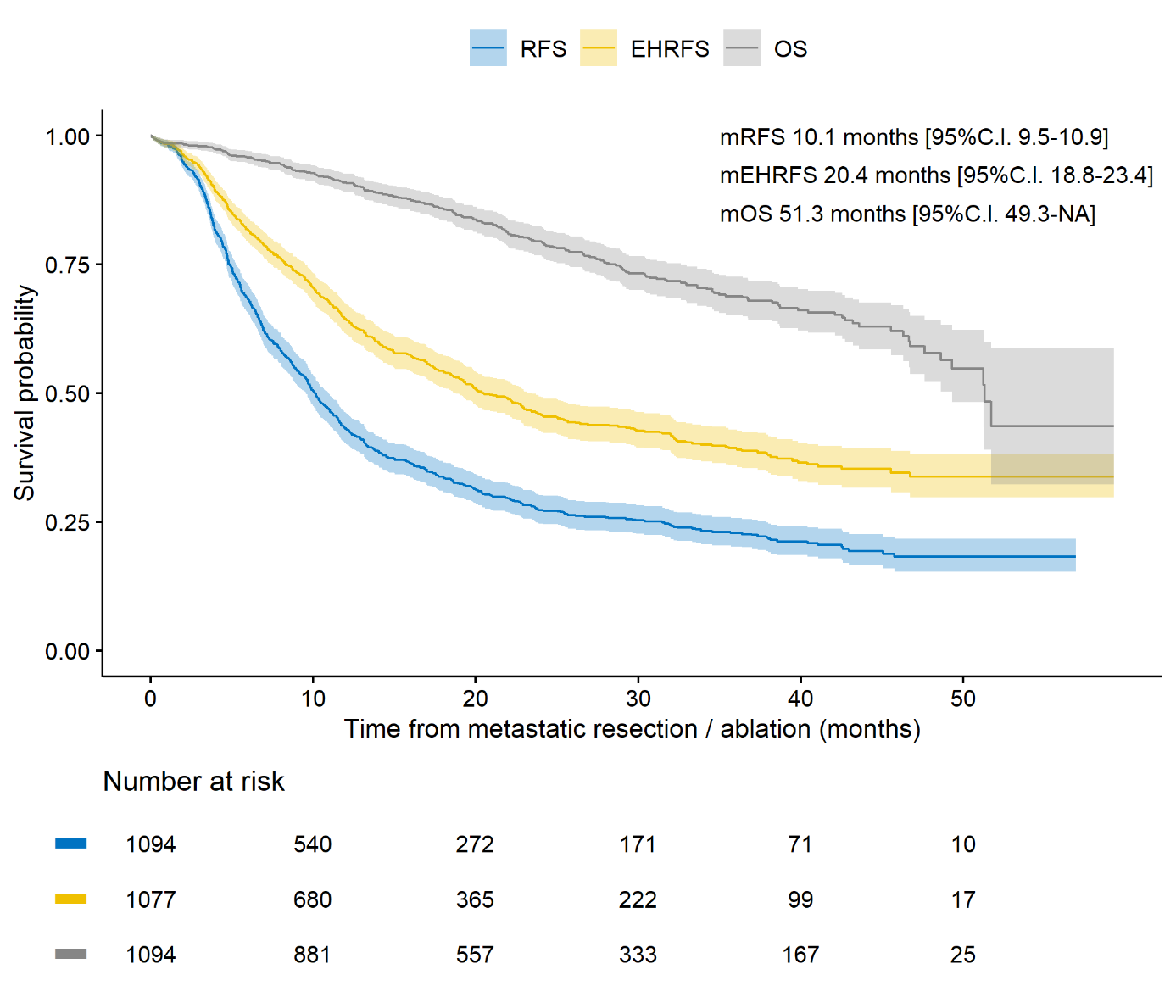


Legend. Kaplan Meier survival curves with 95% confidence intervals are shown for RFS, EHRFS and OS for the whole cohort. The number of patients at risk is indicated in the risk table.

*Abbreviations:* EHRFS (extrahepatic recurrence-free survival), mEHRFS (median EHRFS), mOS (median OS), mRFS (median RFS), OS (overall survival), RFS (recurrence-free survival).

**Table S2.** Detailed information about first recurrence and first extrahepatic recurrence

|  | | **Total cohort**  ***n* = 1105** |
| --- | --- | --- |
|  | | n (%) |
| **RFS data** | |  |
| **Event** | |  |
|  | No | 287 (26.0) |
|  | Yes | 807 (73.0) |
| Recurrence | | 765 |
| Death | | 42 |
| Missing | | 11 (1.0) |
|  | |  |
| **Site of first recurrence,** *n = 765* | |  |
|  | Liver-only | 332 (43.3) |
|  | Extrahepatic  Extrahepatic only  Extrahepatic & intrahepatic | 399 (52.2)  189  210 |
|  | Missing site of recurrence | 34 (4.4) |
|  | |  |
| **EHRFS data** | |  |
| **Extrahepatic event during follow up** | |  |
|  | No | 520 (48.3) |
|  | Yes | 557 (51.7) |
|  | Recurrence | 478 |
|  | Death | 79 |
|  | Missing | 28 (2.5) |
|  | |  |
| **Site of first extrahepatic recurrence,** *n = 478* | |  |
|  | Lung | 213 (44.6) |
|  | Intra-abdominal | 55 (11.5) |
|  | Lymph nodes | 55 (11.5) |
|  | Bone | 5 (1.0) |
|  | Genito-urinary tract | 5 (1.0) |
|  | Soft tissue | 12 (2.5) |
|  | Brain with/without other sites | 6 (1.3) |
|  | Multiple extrahepatic sites | 127 (26.6) |

*Abbreviations:* *n* (count), RFS (recurrence-free survival), EHRFS (extrahepatic recurrence-free survival).

**Figure S2**. Post-recurrence overall survival of patients according to site of extrahepatic recurrence


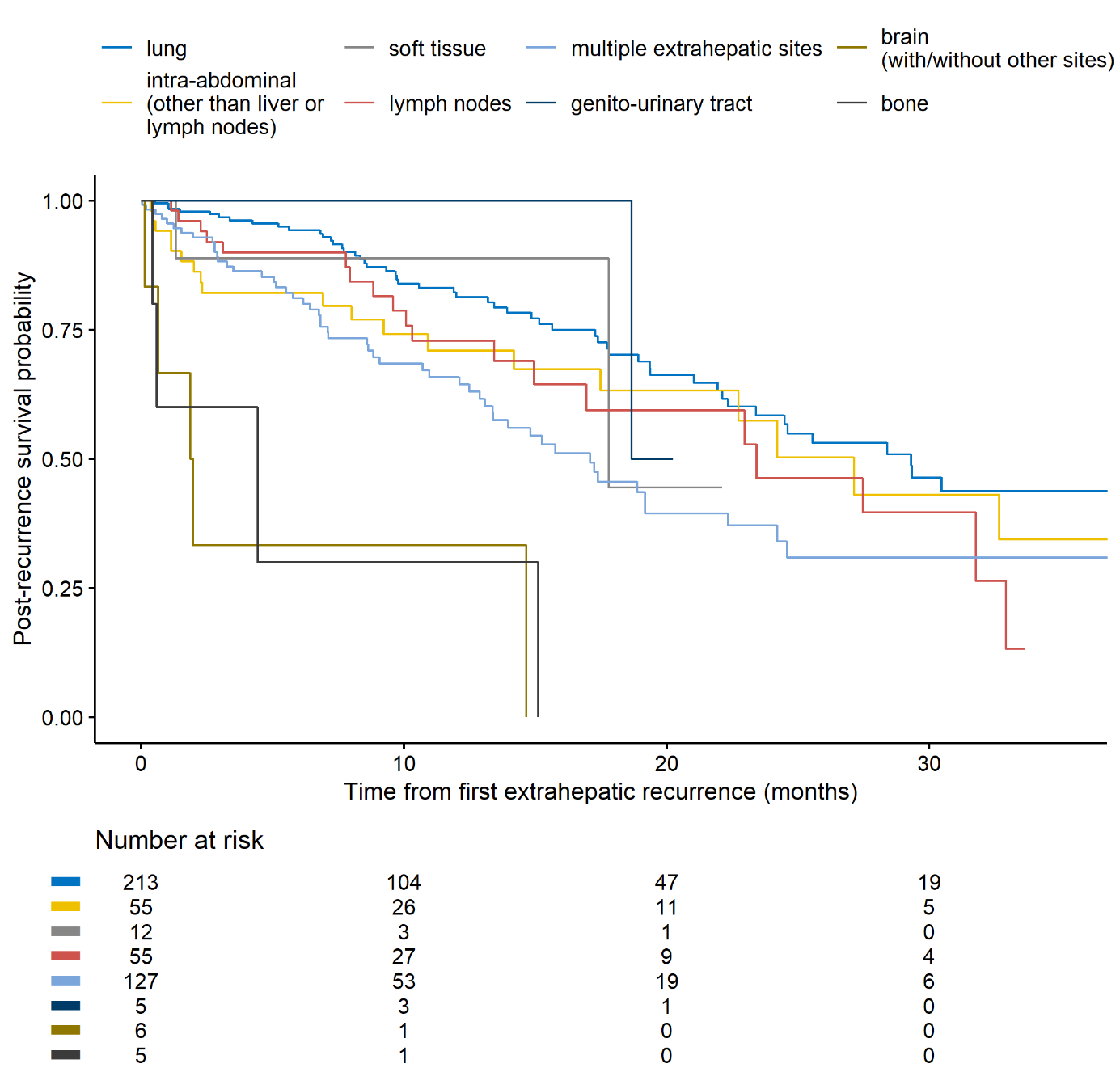


Legend. A KM plot of post-recurrence survival in patients according to the first site of extrahepatic recurrence. Categories indicate the site of extrahepatic metastasis, but may also include hepatic localization. A log-rank test for the post-recurrence survival probability per site of extrahepatic recurrence was performed (*p<*0.0001). A log-rank test for the time to recurrence per site of extrahepatic recurrence was performed (*p*=0.552).

**Figure S3.** Landmark analysis at six months showing Kaplan-Meier curves of patients with extrahepatic, intrahepatic-only and no recurrences within six months


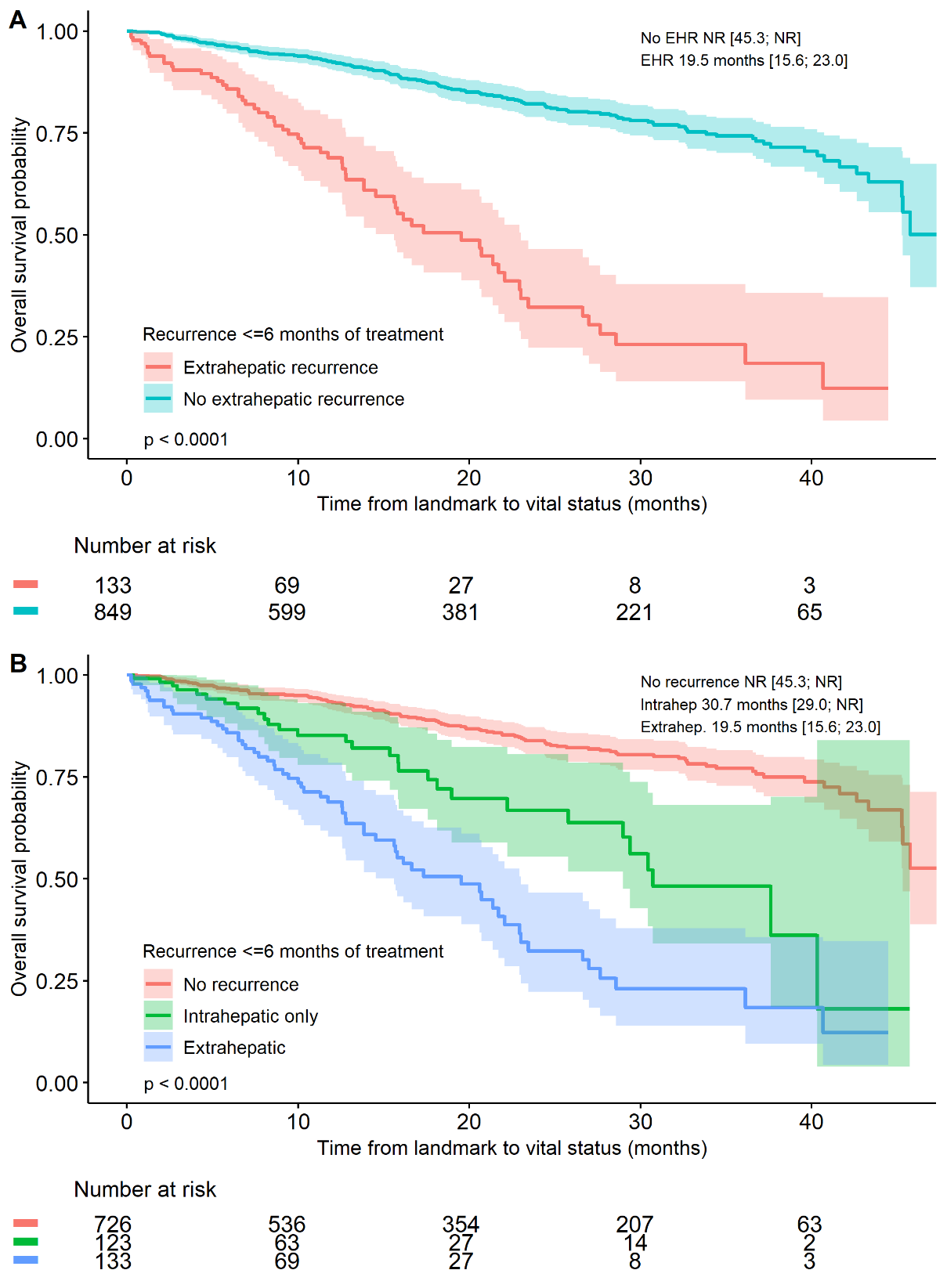


Legend. A landmark analysis Kaplan Meier survival plot is shown, indicating the overall survival of patients after the landmark point (6 months after local treatment of CRLM), according to the site of recurrence which patients had experienced within 6 months of local treatment of CRLM. A. The 2 groups are extrahepatic recurrence (which includes death as an event) versus no extrahepatic recurrence. B. The 3 recurrence site groups are: no recurrence, intrahepatic recurrence only, extrahepatic recurrence (including *n=100* intra + extrahepatic recurrence). The log-rank *p*-value is indicated in the plot along with the observed median survival and 95% confidence intervals.

*Abbreviations:* EHR (extrahepatic recurrence), n (count), NR (not reached).

**Figure S4.** Kaplan-Meier curves describing post-resection overall survival, recurrence-free survival and extrahepatic recurrence-free survival in the total cohort according to location of primary tumour and *RAS/BRAF* mutational status.


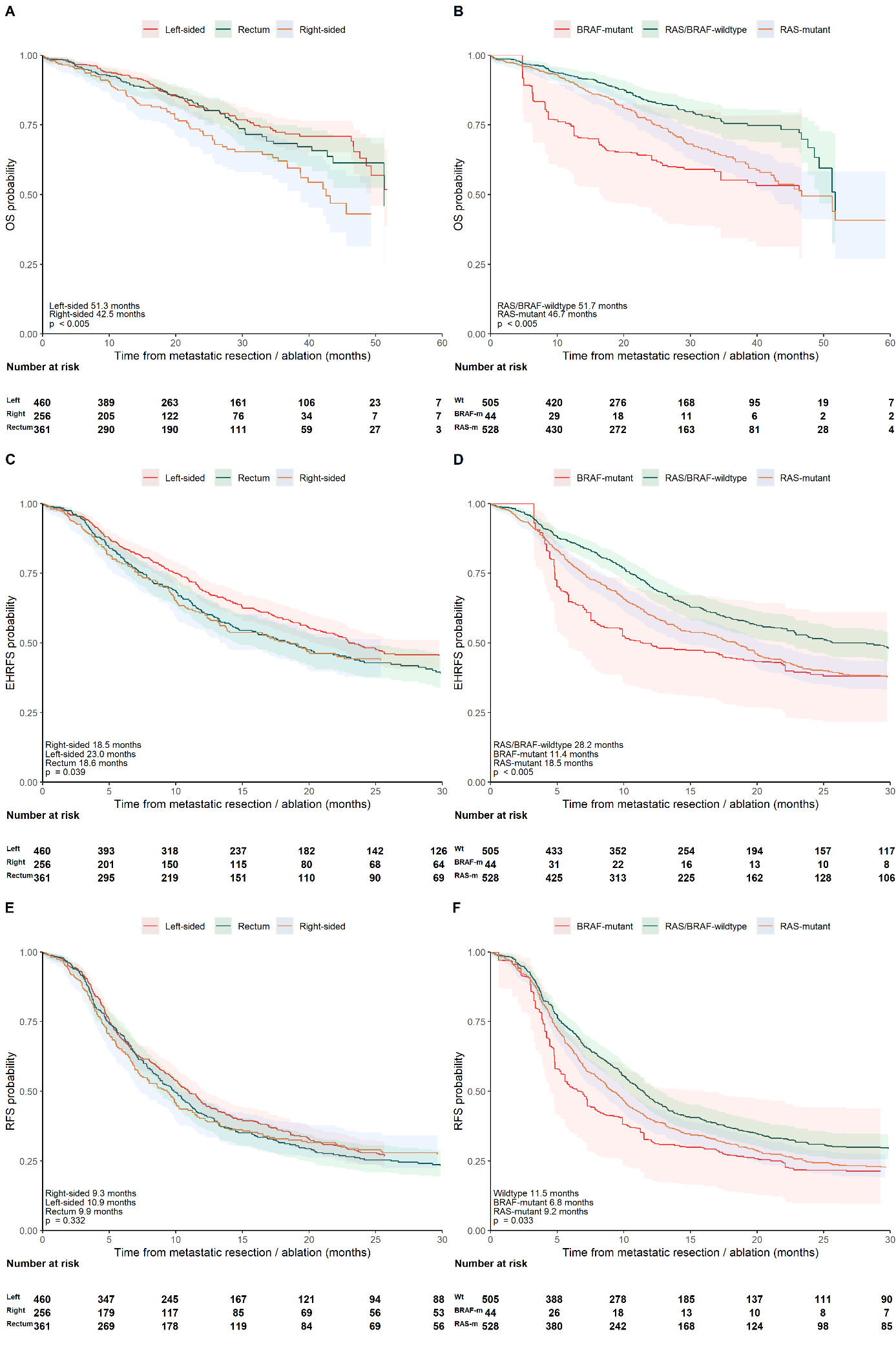


Legend. Kaplan-Meier curves describing post-resection overall survival (A and B), and recurrence-free survival (C and D) and extrahepatic recurrence-free survival (E and F) in the total cohort according to location of primary tumour (A, C, D) and *RAS/BRAF* mutational status (B, D, F) using the imputed dataset. The observed median survival and 95% confidence intervals are indicated in the plot and not indicated if not reached within the follow-up period.

*Abbreviations:* *BRAF*-m (*BRAF*-mutant), EHRFS (extrahepatic recurrence-free survival), mEHRFS (median EHRFS), mOS (median OS), mRFS (median RFS), OS (overall survival), *RAS*-m (*RAS*-mutant), RFS (recurrence-free survival), Wt (*RAS/BRAF*-wildtype).

**Figure S5.** Hazard ratio for EHRFS for continuous variables modeled non-linearly using restricted cubic splines


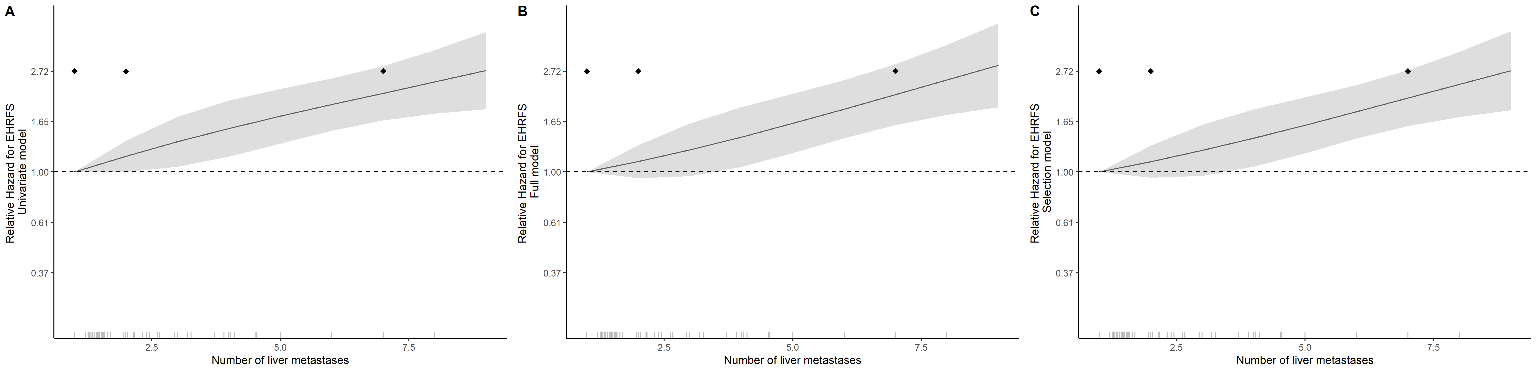

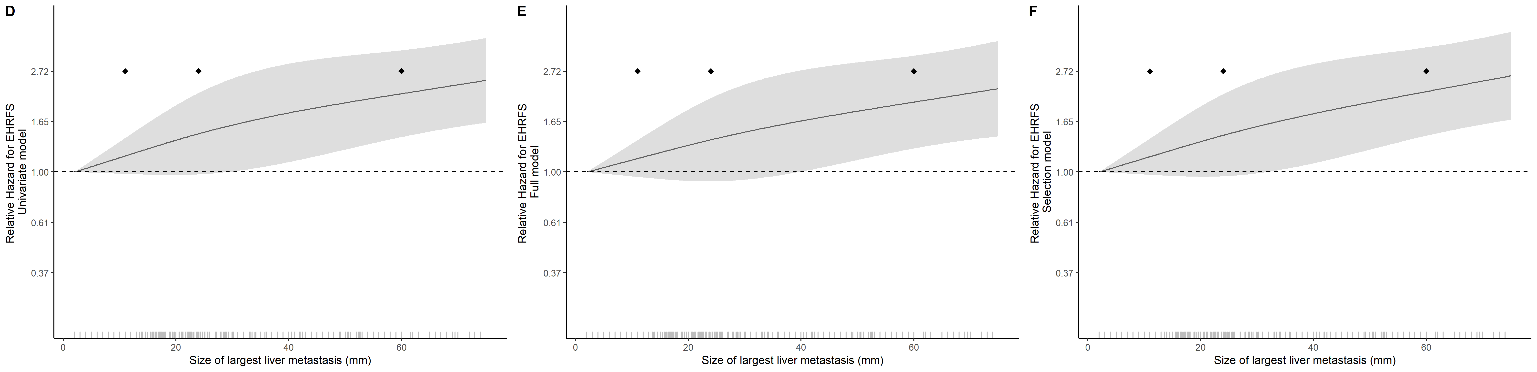

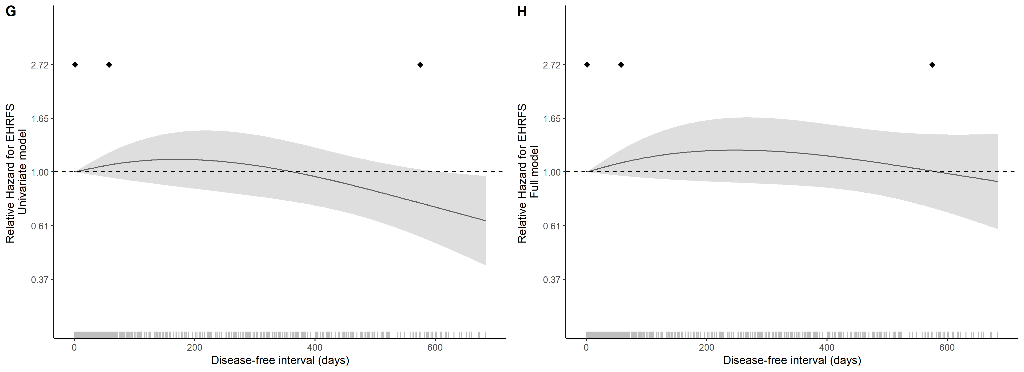

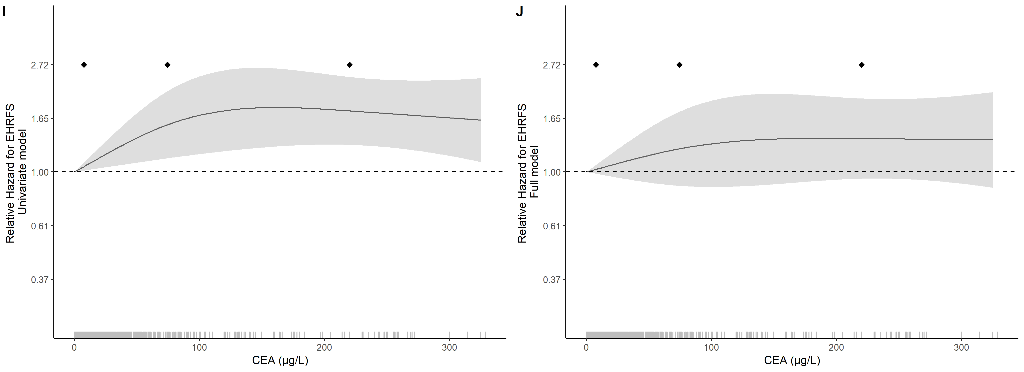


Legend. The pooled relative hazard and 95% confidence interval ribbon for EHRFS within 12 months from local treatment of CRLM according to one of three models (univariable, full multivariable and selection multivariable) are shown in the y-axis relative to the observed value of the continuous variable plotted on the x-axis. Each column indicates a model type with the univariable results in plots A, D, G, I; the full multivariable results in plots B, E, H, J; and the selection multivariable results in plots C and F (disease-free interval and pre-operative CEA were not included in the selection model). Each row shows a continuous variable, with number of liver metastases (A – C), size of largest liver metastasis (D – F), disease-free interval (G-H) and pre-operative CEA values (I-J). All 4 continuous variables were modeled using restricted cubic splines analysis with 3 knot positions (positions are indicated by the dots in the plot). The frequency of the observed values are indicated along the x-axis.

*Abbreviations:* CEA (carcinoembryonic antigen), CRLM (colorectal liver metastasis), EHRFS (extrahepatic recurrence-free survival), mm (millimeter), μg/L (microgram per liter).

**Figure S6.** Time-dependent ROC curve for the EHRFS model indicating the true positives and false positives 6 and 12 months after local treatment for CRLM


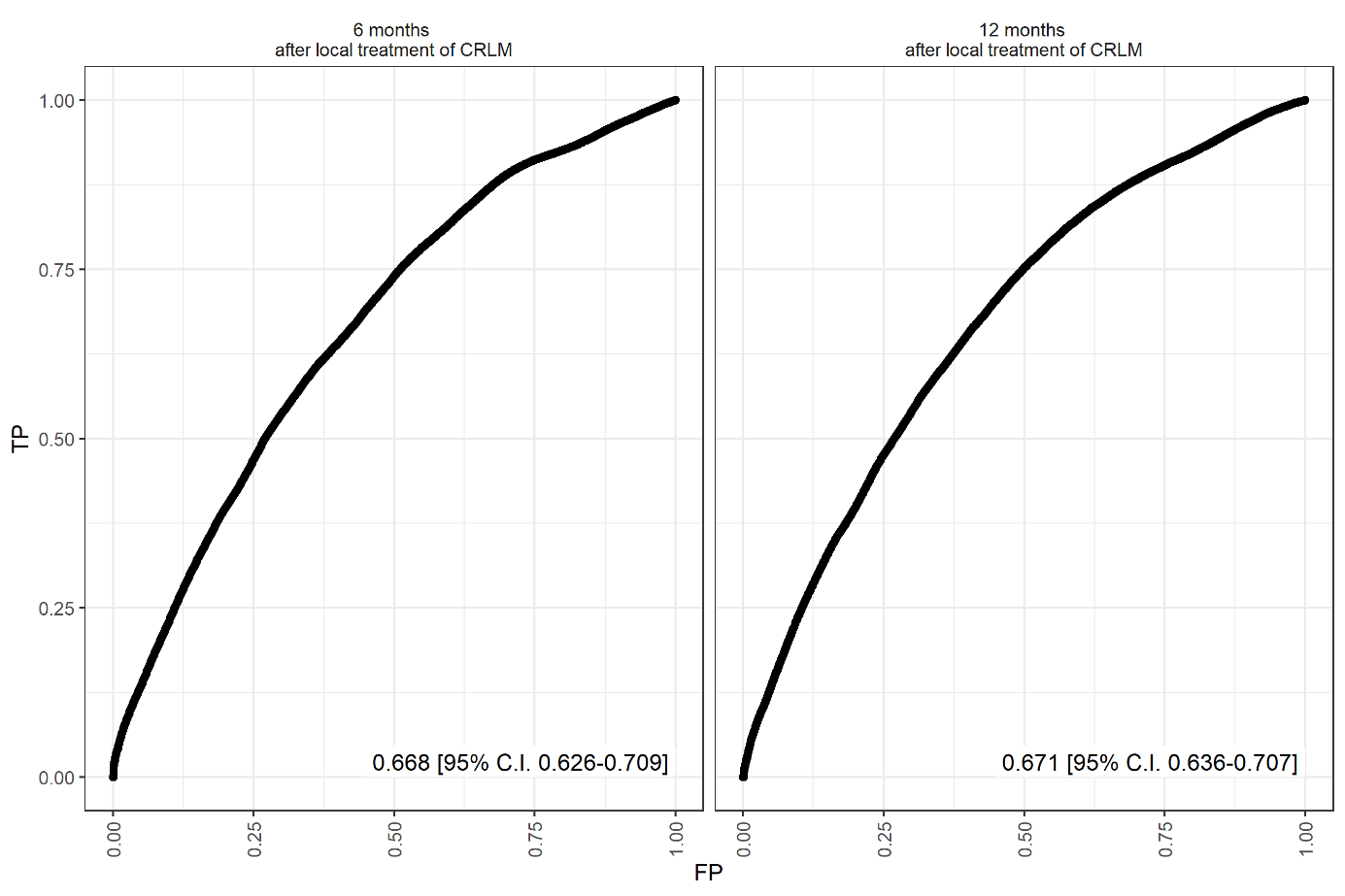


Legend. In a cumulative case/ dynamic control ROC analysis, the time-dependent receiver operator curve is shown with on the y-axis the true positive and the x-axis the false positive based on the model’s linear predictor for each individual compared to the observed EHRFS at the given timepoint. The plot indicates how well the model predicts the survival time for the patients for 6 and 12 months after local treatment for CRLM, respectively. The AUC with 95% C.I. is indicated in each plot. The confidence intervals for AUC were calculated using 1000 bootstrap samples.

*Abbreviations:* AUC (area under the (ROC) curve), C.I. (confidence interval), CRLM (colorectal liver metastasis), FP (false positive), ROC (receiver operator curve), TP (true positive).

**Figure S7.** Internal-external cross-validation results


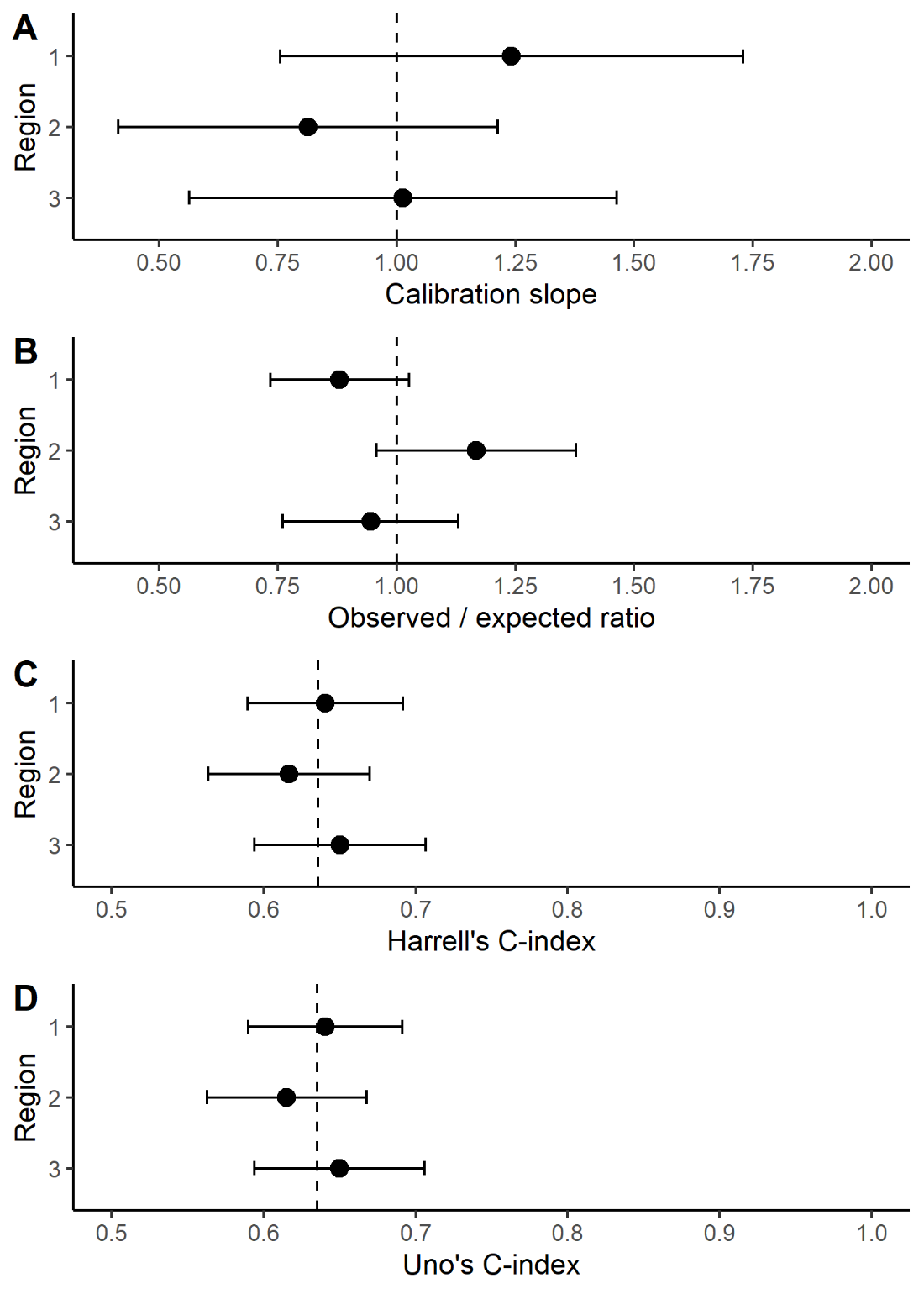


Legend. The results for internal-external cross-validation are shown, including the calibration slope (A), observed/expected ratio (B), Harrell’s C-index (C) and Uno’s C-index (D). We used internal-external cross-validation to evaluate the generalizability of the model. The data were split in three geographic regions and all modeling steps including backward selection of variables and internal validation were repeated in two of three regions, after which the performance of the overfitting-adjusted model was evaluated in the left-out geographical region (C-index, calibration slope and intercept). Each geographic region was left-out of model development once, resulting in three estimates of external validation. The reference line in plot C & D indicates the mean C-index.

*Abbreviations:* C-index (calibration-index).

**References**

1 Malik HZ, Gomez D, Wong V, Al-Mukthar A, Toogood GJ, Lodge JPA, *et al.* Predictors of early disease recurrence following hepatic resection for colorectal cancer metastasis. *Eur J Surg Oncol*. 2007; **33**: 1003–1009.

2 Vigano L, Capussotti L, Lapointe R, Barroso E, Hubert C, Giuliante F, *et al.* Early recurrence after liver resection for colorectal metastases: risk factors, prognosis, and treatment. A LiverMetSurvey-based study of 6,025 patients. *Ann Surg Oncol*. 2014; **21**: 1276–1286.

3 Riley RD, Snell KIE, Ensor J, Burke DL, Harrell FE, Moons KGM, *et al.* Minimum sample size for developing a multivariable prediction model: PART II - binary and time-to-event outcomes. *Stat Med*. 2019; **38**: 1276–1296.

4 Chen Y, Chang W, Ren L, Chen J, Tang W, Liu T, *et al.* Comprehensive Evaluation of Relapse Risk (CERR) Score for Colorectal Liver Metastases: Development and Validation. *Oncologist*. 2020; **25**: e1031–e1041.

5 Fong Y, Fortner J, Sun RL, Brennan MF, Blumgart LH. Clinical score for predicting recurrence after hepatic resection for metastatic colorectal cancer: analysis of 1001 consecutive cases. *Ann Surg*. 1999; **230**: 309–321.

6 Margonis GA, Sasaki K, Gholami S, Kim Y, Andreatos N, Rezaee N, *et al.* Genetic And Morphological Evaluation (GAME) score for patients with colorectal liver metastases. *Br J Surg*. 2018; **105**: 1210–1220.

7 Paredes AZ, Hyer JM, Tsilimigras DI, Moro A, Bagante F, Guglielmi A, *et al.* A Novel Machine-Learning Approach to Predict Recurrence After Resection of Colorectal Liver Metastases. *Ann Surg Oncol*. Springer International Publishing; 2020; **27**: 5139–5147.

8 Brudvik KW, Jones RP, Giuliante F, Shindoh J, Passot G, Chung MH, *et al.* RAS Mutation Clinical Risk Score to Predict Survival After Resection of Colorectal Liver Metastases. *Ann Surg*. 2019; **269**: 120–126.

9 Buisman FE, Galjart B, Buettner S, Groot Koerkamp B, Grunhagen DJ, Verhoef C. Primary tumor location and the prognosis of patients after local treatment of colorectal liver metastases: a systematic review and meta-analysis. *HPB*. 2020; **22**: 351–357.

10 Yamashita S, Chun YS, Kopetz SE, Vauthey JN. Biomarkers in colorectal liver metastases. *Br J Surg*. 2018; **105**: 618–627.

11 van Buuren S, Groothuis-Oudshoorn K, Vink G, Doove L, Jolani S, Schouten R, *et al.* mice: Multivariate Imputation by Chained Equations in R. R package. *J Stat Softw*. 2011; **45**: 1–67.

12 Sasaki K, Morioka D, Conci S, Margonis GA, Sawada Y, Ruzzenente A, *et al.* The Tumor Burden Score: A New ‘Metro-ticket’ Prognostic Tool For Colorectal Liver Metastases Based on Tumor Size and Number of Tumors. *Ann Surg*. 2018; **267**: 132–141.

13 Nordlinger B, Sorbye H, Glimelius B, Poston GJ, Schlag PM, Rougier P, *et al.* Perioperative FOLFOX4 chemotherapy and surgery versus surgery alone for resectable liver metastases from colorectal cancer (EORTC 40983): long-term results of a randomised, controlled, phase 3 trial. *Lancet Oncol*. 2013; **14**: 1208–1215.

14 Kanemitsu Y, Shimizu Y, Mizusawa J, Inaba Y, Hamaguchi T, Shida D, *et al.* A randomized phase II/III trial comparing hepatectomy followed by mFOLFOX6 with hepatectomy alone for liver metastasis from colorectal cancer: JCOG0603 study. *J Clin Oncol*. 2020; **38**: 4005.

15 Steyerberg EW, Harrell FE. Prediction models need appropriate internal, internal-external, and external validation. *J Clin Epidemiol*. 2016; **69**: 245–247.

16 Musoro JZ, Zwinderman AH, Puhan MA, Ter Riet G, Geskus RB. Validation of prediction models based on lasso regression with multiply imputed data. *BMC Med Res Methodol*. 2014; **14**: 1–13.

17 Rousson V, Zumbrunn T. Decision curve analysis revisited: Overall net benefit, relationships to ROC curve analysis, and application to case-control studies. *BMC Med Inform Decis Mak*. 2011; **11**: 1–9.

18 Takeda Y, Mise Y, Takahashi Y, Ito H, Inoue Y, Yoshioka R, *et al.* Limited Prognostic Value of KRAS in Patients Undergoing Hepatectomy for Colorectal Liver Metastases. *Ann Surg Oncol*. 2022; **29**: 2383–2391.

19 Kobayashi S, Takahashi S, Takahashi N, Masuishi T, Shoji H, Shinozaki E, *et al.* Survival Outcomes of Resected BRAF V600E Mutant Colorectal Liver Metastases: A Multicenter Retrospective Cohort Study in Japan. *Ann Surg Oncol*. 2020; **27**: 3307–3315.
